# Predicting the Need to Visit a Dentist in Young to Middle-Aged Adults: A Development and External Validation

**DOI:** 10.3390/dj14070398

**Published:** 2026-07-01

**Authors:** Miika Penttala

**Affiliations:** Department of Oral and Maxillofacial Diseases, Head and Neck Center, University of Helsinki and Helsinki University Hospital, 00290 Helsinki, Finland; miika.penttala@helsinki.fi

**Keywords:** external validation, logistic regression, NHANES, oral health, predictive modelling, screening, young to middle-aged adults

## Abstract

**Background/Objectives:** A multivariable prediction model was developed and externally validated to estimate an individual’s current need to visit a dentist among young to middle-aged adults. The objective was to provide an accessible, non-invasive screening tool for independent home self-assessment or integration within routine healthcare workflows. **Methods:** A cross-sectional study utilised data from two National Health and Nutrition Examination Survey (NHANES) cycles (2011–2014). Logistic regression was applied to develop the prediction model among 1870 dentate adults aged 30–50, a critical onset period for oral diseases. The primary outcome was derived from objective oral examinations conducted by licensed dentists. The resulting questionnaire-based tool provides two recommendations: a visit to a dentist or continuation of routine oral care. **Results:** External validation using the independent NHANES 2013–2014 cohort (n = 2024) demonstrated robust and clinically relevant predictive capacity, with an AUC of 0.822 (95% CI 0.803–0.842). The model exhibited acceptable calibration (slope 0.85; intercept 0.04) and stable operation. Decision-curve analysis showed net benefit across thresholds; at Pt = 0.33, 14/100 unnecessary screenings were avoided compared with treat-all. Internal validation using a 10% hold-out partition (n = 184) supported these findings, indicating similarly strong discrimination (AUC 0.817, 95% CI 0.751–0.884). **Conclusions:** Early midlife is a consistent and informative period for oral health intervention. A scalable 14-predictor architecture using accessible indicators established high clinical utility, supporting integration into digital health and primary care. This approach enables efficient population-level prevention. Large-scale, nationally representative NHANES data allowed for the investigation of less-studied factors, including intermittent smoking, metabolic dysregulation, and elevated cholesterol levels.

## 1. Introduction

Oral diseases are among the most common non-communicable conditions worldwide and contribute substantially to population-level morbidity. The impact of oral conditions extends well beyond the oral cavity. Periodontitis shares a well-established bidirectional relationship with diabetes, where systemic inflammation actively impairs glycaemic control [1]. Furthermore, chronic periodontal inflammation is independently linked to an increased risk of atherosclerotic cardiovascular diseases through shared inflammatory pathways and metabolic dysregulation [2]. Global Burden of Disease analyses confirm that these oral conditions, particularly untreated caries and periodontal disease, remain highly prevalent across adulthood and contribute substantially to the global disability burden [3,4]. Periodontitis affects nearly half of adults over 30 years [5], with incidence rising markedly in the late fourth decade [6], coinciding with increasing rates of diabetes and other systemic comorbidities [1,2].

Given that the need to visit a dentist is frequently overlooked both by individuals and during routine health checks, scalable risk-stratification tools deployable at home, within primary care, or across occupational health settings are needed to identify those requiring timely professional assessment. To address this gap, a predictive model utilising NHANES data was developed for a mobile application interface to estimate clinically verified objective treatment and preventive needs. By anchoring the target output strictly in independent, standardised clinical examinations performed by licensed dentists, the approach minimises the subjective bias and variability of perceived need, aiming to support earlier intervention among young to middle-aged adults, when preventive action can most effectively mitigate long-term oral and systemic decline.

Utilising the NHANES 2011–2014 dataset and its gold-standard oral health examination protocol [7], this study addresses a critical gap by focusing on adults aged 30–50 years. While pediatric and geriatric oral health burdens are well characterised in global epidemiological studies [3,4], early-to-mid adulthood represents a pivotal window in which timely intervention can prevent progression to irreversible tissue damage [8]. Despite the persistent high burden of oral conditions among younger adult populations [3,4], existing prediction models have historically not been tailored to age-specific risk profiles [9], and there is currently a lack of accepted or standardised models that can reliably identify at-risk individuals across different populations [10]. Whereas existing risk assessment tools frequently rely on clinical measurements or laboratory biomarkers, the proposed model offers a non-invasive, scalable alternative by utilising non-clinical, user-friendly questions.

The objective of this study was to develop an easy-to-use prediction model trained to estimate the need to visit a dentist based on clinical examinations conducted by licensed dentists at the time of assessment, and to externally validate its performance within a representative sample of middle-aged adults. Evidence is provided to support population-level screening protocols, demonstrating how readily accessible predictors can be leveraged for integration into digital health platforms and routine healthcare pathways.

## 2. Materials and Methods

### 2.1. Study Design and Data Source

This cross-sectional study drew on data from the NHANES 2011–2014 cycles, integrating clinical, laboratory, and behavioral measures [7]. Participants’ oral health information was collected using standardised clinical protocols, including full-mouth periodontal examinations, caries assessments, and oral hygiene markers [7]. In addition, self-reported questionnaires, anthropometric measures, and systemic health markers were included. The 2011–2012 cycles served as the development cohort, while 2013–2014 provided independent temporal validation [11]. To minimise incorporation bias, strict methodological and procedural separation was maintained within the NHANES protocol. Subjective predictors were collected independently during prior household interviews, ensuring that clinical examiners in the Mobile Examination Center remained fully blinded to these responses during the objective oral examinations [7,12,13]. Furthermore, examiner-participant interaction was strictly standardised; clinicians were procedurally restricted from conducting unstandardised history interviewing, and any verbal communication was limited to mandatory medical screenings and predetermined study protocols [12,13].

NHANES is a nationally representative survey of the US civilian population, conducted using mobile examination centers (MECs). The original survey protocol was approved by the NCHS Research Ethics Review Board (Protocol #2011-17; approval date: 10 November 2011), and this secondary analysis of de-identified data was deemed exempt from further institutional oversight.

To predict individuals’ need to visit the dentist, NHANES Overall recommendation for care (OHAREC) variable was utilised. In the recommendation, licensed dentists assigned each participant to one of four standardised referral categories: (1) see a dentist immediately, (2) see a dentist within 2 weeks, (3) see a dentist at earliest convenience, or (4) continue regular routine care (NHANES variable OHAREC, located in the OHXREF_G data file [14]). These four categories were dichotomised in this study, with 1 indicating the need to visit a dentist and 0 indicating continuation of regular oral care. The variable represents an expert clinical synthesis of the research participant’s oral health status. User interaction requirements of the prediction model are detailed in Supplementary Materials: Note S1.

The overall oral care recommendations in the 2011–2012 cohort were overwhelmingly driven by three key factors: decayed teeth, gum disease or problems, and oral hygiene status. These categories encompass specific clinical markers such as coronal caries, indicators of periodontal disease such as gingival inflammation or attachment loss, and the accumulation of visible plaque or calculus. Only a small share of triggers arose from other clinically significant findings, such as soft-tissue abnormalities (including ulcers, lesions, or suspicious mucosal changes), failing restorations, dental trauma, and acute or urgent conditions. The robustness of these trigger categories, which form the Overall recommendation for care variable, and related descriptive statistics, are evaluated in Supplementary Materials (Note S2 and Figure S1). A brief methodological commentary in Note S2 also addresses the omission of root caries in this cohort and explains why its impact on the predicted outcome is minimal.

The core NHANES oral health measures used in this study demonstrated robust examiner reliability for epidemiologic use. Assessments of decayed teeth (dental caries) and tooth count showed excellent inter-examiner agreement (κ > 0.80), and periodontal measurements achieved moderate to substantial reliability (κ ≈ 0.55–0.75) [7,12,13]. Furthermore, continuous periodontal measures, such as clinical attachment loss and pocket depth, demonstrated excellent agreement with inter-class correlation coefficients (ICCs) ranging from 0.79 to 0.90 [7]. Indicators of hygiene status—based on clinical assessments of oral cleanliness—have demonstrated acceptable reproducibility within the NHANES calibration framework [7,15]. All criteria followed the standardised operational definitions and examination protocols outlined in the NHANES Oral Health Examiners Manual [12,13]. These findings indicate that the clinical measures underlying the predicted outcome in this study meet established thresholds for reliable population-level assessment. The study adhered to STROBE guidelines for cross-sectional research [16] and followed the TRIPOD+AI recommendations for developing and validating clinical prediction models [17].

### 2.2. Participant Selection and Variables

**Figure 1:**
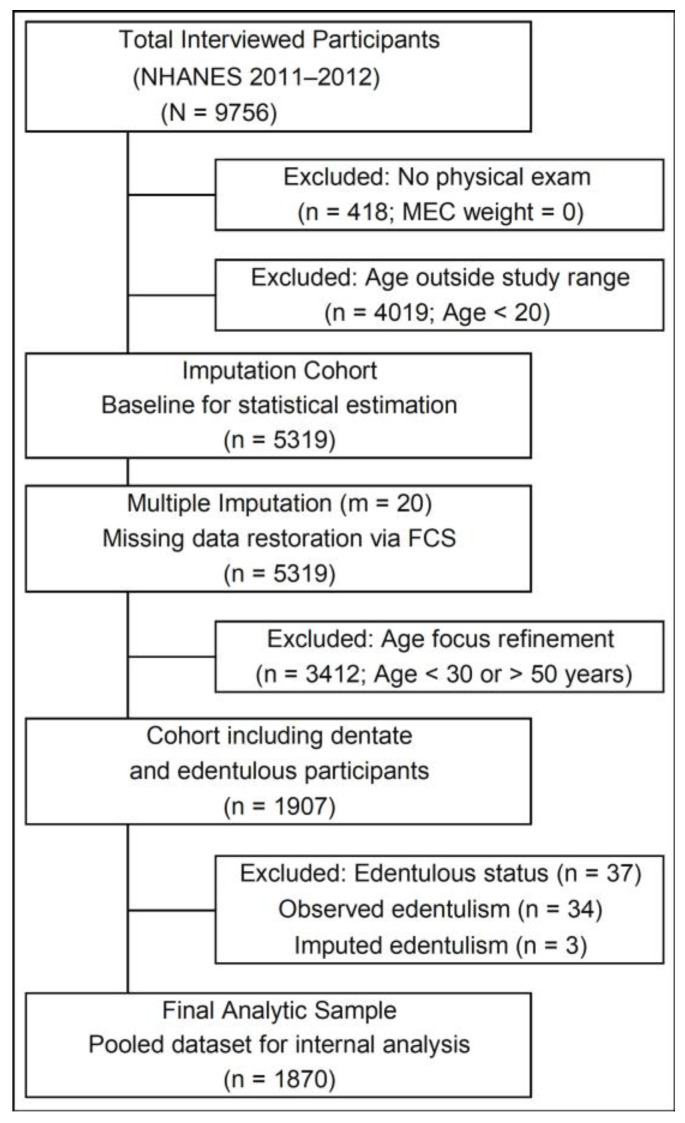
Participant selection and data restoration flowchart. The diagram shows the progression from initial NHANES 2011–2012 recruitment to the final analytic sample. Multiple imputation (m = 20) was applied to eligible participants (n = 5319). After restricting to adults aged 30–50 years and applying biological eligibility criteria (dentate status, >0 teeth), the restored analytic sample comprised 1870 participants.

The final regression modeling sample was restricted to dentate adults aged 30–50 years with non-zero Mobile Examination Center weights (WTMEC2YR). No participants were excluded due to missing data, because multiple imputation was incorporated into the study design. Dentate status was defined as possessing ≥1 erupted permanent tooth (excluding third molars), and individuals without any erupted permanent teeth were classified as edentulous and excluded during the final stage of participant selection (Figure 1). Importantly, edentulous individuals were not excluded prior to multiple imputation to prevent selection bias and avoid the premature deletion of participants with missing dental exam metrics. This sequencing allowed the imputation model to utilise the full correlation structure of the cohort and enabled the post-imputation identification and exclusion of individuals with imputed edentulism (Figure 1).

The multivariable framework incorporated 14 preselected predictors, chosen for their established roles in the oral health context. By selecting these variables manually—rather than relying on automated stepwise procedures—it was ensured that the model remained clinically actionable and provided a robust basis for evaluating how diverse health profiles influence an individual’s need to visit a dentist.

Building on this conceptual foundation, the model is optimised for clinical utility through a streamlined input architecture. Of the 14 predictors, 11 are simple multiple-choice questions, while only three continuous variables—age, waist circumference, and the ratio of family income to poverty (PIR)—require numeric entry. Crucially, PIR is simplified for routine practice: users can instantly determine it by cross-referencing household size and income brackets (see Table S1 in Supplementary Materials: Quick Estimation Guide). By automating complex risk calculations behind this user-friendly interface, the model provides high point-of-care utility without requiring specialised diagnostics. For implementation details regarding data quality, see Supplementary Materials: Note S3.

### 2.3. Statistical Analysis

#### 2.3.1. Data Restoration and Multiple Imputation

Missing data were addressed using Multiple Imputation (m = 20) with Fully Conditional Specification and Predictive Mean Matching in IBM SPSS Statistics (version 31.0) [18]. Imputation was performed on the initial NHANES 2011–2012 cohort (n = 5319), with missingness patterns summarised in Supplementary Materials: Table S2. The imputation model included all 14 predictors, the binary outcome, and five auxiliary variables (HbA1c, systolic blood pressure [SBP], total cholesterol, body mass index [BMI], and tooth count) to support the Missing at Random (MAR) assumption. These auxiliary variables were used solely for imputation and excluded from the final regression model. The external validation cohort (NHANES 2013–2014) underwent identical preprocessing and was imputed as a separate, independent stream to prevent data leakage.

#### 2.3.2. Cohort Partitioning and Validation Strategy

To ensure reproducibility and maintain a transparent and prespecified validation structure, the source population was partitioned into a 10% internal validation subset (n = 184) and a 90% model-development cohort (n = 1686) using a deterministic transformation of the participant sequence number (SEQN). Further methodological details and justification for this approach are provided in Supplementary Materials: Note S4. These assignments were held constant across all 20 imputations. The analytic cohort was restricted to adults aged 30–50 years, yielding a final dentate sample of n = 1870 after excluding edentulous participants (Figure 1). Missingness patterns for this subset are reported in Supplementary Materials (Table S3). External validation used the NHANES 2013–2014 cohort (n = 2024) to assess model transportability by applying pooled coefficients and the intercept from the training set to this independent sample (Supplementary Materials: Note S5). Because the external cohort served as the primary and decisive validation dataset, the internal 10% subset was used only as a secondary, supportive check of model consistency.

#### 2.3.3. Survey Weighting and Normalisation

To preserve national representativeness, the original MEC weights were incorporated into the imputation process. During the regression analyses, weights were normalised within each imputed dataset by dividing the original MEC weights by the mean weight of that dataset. Methodological details and sample sizes are provided in Supplementary Materials (Note S6). This normalisation anchored the effective sample size to the observed case count and prevented inflation of statistical significance while maintaining relative population proportions. All weighting procedures consistently relied on the normalised weights to ensure comparability across analyses [19,20,21].

#### 2.3.4. Multivariable Model Development

Multivariable logistic regression was performed using forced entry of 14 prespecified predictors. Pooled coefficients, adjusted odds ratios (aOR), and 95% confidence intervals (CI) were obtained using Rubin’s Rules. The model maintained an events-per-variable ratio well above recommended thresholds for logistic regression [22]. Linearity of continuous predictors was assessed using Box–Tidwell tests and visual inspection. Categorical variables were dummy-coded using the lowest-risk category as reference; this included multi-level variables, which were expanded into the appropriate number of indicator terms (Supplementary Materials: Table S4). Standard modelling principles for logistic regression were followed throughout [23]. Full model specification is provided in Supplementary Materials: Note S7.

#### 2.3.5. Model Evaluation

All performance-related standard errors (SEs) and 95% confidence intervals (CIs) were pooled across the 20 imputed datasets using Rubin’s Rules, incorporating both within- and between-imputation variance.

Discrimination was assessed using the C-statistic (AUC) and the Brier score. AUC SEs were obtained using the non-parametric Hanley–McNeil method, and Brier score uncertainty was quantified using the standard error of the mean (SEM) of squared individual-level residuals.

Sensitivity and specificity were calculated within each imputed dataset and pooled using Rubin’s Rules, with 95% CIs derived from the standard error of a proportion (*p*(1 − *p*)/*n*), reflecting their binomial nature.

Calibration was evaluated using pooled decile-level comparisons of predicted and observed event rates, visualised with calibration plots. Uncertainty was quantified using the SEM, calculated as the standard deviation of observed proportions within each decile divided by √n.

Clinical utility was assessed using Decision Curve Analysis (DCA), comparing net benefit with treat-all and treat-none strategies. Net benefit and its 95% CIs were estimated using the Delta method applied to the decision-analytic net benefit function, utilising the weighted analytic sample size to ensure consistency with prevalence and test characteristics, and pooled using Rubin’s Rules.

In addition, predictor contributions were summarised using Δ Nagelkerke R^2^ for training and external datasets. Threshold behaviour was examined across imputations to assess the stability of Youden-optimal cut-offs (Supplementary Materials: Note S8).

## 3. Results

### 3.1. Sample Characteristics and Imputation Performance

The derivation cohort comprised 1870 dentate adults aged 30–50 years. Thirty-four individuals were excluded due to confirmed edentulism, and an additional mean of three participants (range 0–6) were excluded across the 20 imputation cycles based on predicted edentulism. This resulted in minor fluctuations in analytic sample size (n = 1867–1873), which were accounted for using Rubin’s Rules. To maintain statistical efficiency and ensure consistent variance estimation, pooled survey weights were normalised to the target analytic cohort size of n = 1870.

Imputation quality was evaluated by comparing pooled estimates with observed values to ensure distributional fidelity. Agreement was high: mean absolute differences were below 1.5 percentage points for categorical variables and under 4.3% for continuous measures. This approach restored the intended cohort structure and mitigated the selection bias inherent in complete-case analysis.

The prevalence of a need to visit a dentist was 49.3% in the imputed cohort, calculated as the pooled estimate across the 20 completed datasets, and identical to the complete-case estimate (both calculated using normalised weights). When NHANES examination weights are normalised, the analytic sample remains representative of the non-institutionalised dentate U.S. population aged 30–50 years, supporting the generalisability of the findings (Table 1).

### 3.2. Model Diagnostics and Assumptions

Model diagnostics indicated that the multivariable framework was stable and met the assumptions. Multicollinearity was assessed across all 20 imputed datasets. Variance Inflation Factors (VIF) for the predictors ranged from 1.053 to 1.739 across imputations, with all values remaining well below the conservative threshold of 2.5, indicating minimal multicollinearity. Case-wise influence screening showed that while a small proportion of observations (1.8%) had standardised residuals > 3.0 and only <0.03% had deviance residuals > 3.0, the maximum Cook’s Distance (0.3) remained well below the conventional threshold of 1.0, indicating that no single case exerted undue influence on the model estimates. The linearity of the logit for all continuous predictors was supported by the Box–Tidwell test (*p* > 0.05 for all interaction terms). The model was developed with an events-per-variable ratio of 27 (822 events in the training set), substantially exceeding the recommended minimum of 10 [22].

### 3.3. Multivariable Model Dynamics and Predictor Performance

In the training cohort (n = 1686), Last visit to the dentist accounted for the largest unique variance (ΔR^2^ = 0.030, aOR 1.4–3.5), while Rate your teeth and gums showed a clear dose–response pattern, peaking at a 4.5-fold increase in risk (aOR 4.5, 95% CI 1.9–10.6). Waist circumference and the family income-to-poverty ratio remained highly stable predictors (*p* = 0.001, *p* < 0.001). A notable suppression effect was observed for age: its predictive power intensified from marginal significance (*p* = 0.042) to highly significant (*p* < 0.001) upon adjustment (aOR 1.042; Table 2).

Smoking status remained a primary driver, though its predictive weight shifted after adjustment. While the risk for intermittent smokers remained high and stable (cOR 3.3; aOR 3.3), the effect for daily smokers was notably attenuated (cOR 4.9; aOR 2.8). In parallel, gender emerged as a robust and independent predictor, with males having over double the odds of the outcome (aOR 2.3; *p* < 0.001).

Metabolic indicators added further nuance; borderline diabetes showed a consistent risk trend, although it did not reach statistical significance after adjustment (cOR 2.2, *p* = 0.035; aOR 2.4, *p* = 0.064). In contrast, the association for self-reported diabetes was largely attenuated after adjustment (aOR 1.09; *p* = 0.790). Additionally, awareness of high cholesterol shifted from a neutral crude association (cOR 0.96) to a non-significant protective signal (aOR 0.75; *p* = 0.106) in the multivariable model.

Race and education were significant predictors of the outcome. Compared with Non-Hispanic Whites, all ethnic minority groups showed significantly higher adjusted odds (aORs 1.97–2.84; *p* ≤ 0.011). Similarly, lower educational attainment was associated with increased risk; compared with college graduates, individuals with a high school/GED level (aOR 1.56; *p* = 0.035) or a 9–11th grade education (aOR 1.85; *p* = 0.028) exhibited significantly higher odds, although the effect for the lowest education category did not reach significance after adjustment (*p* = 0.180).

**Figure 2:**
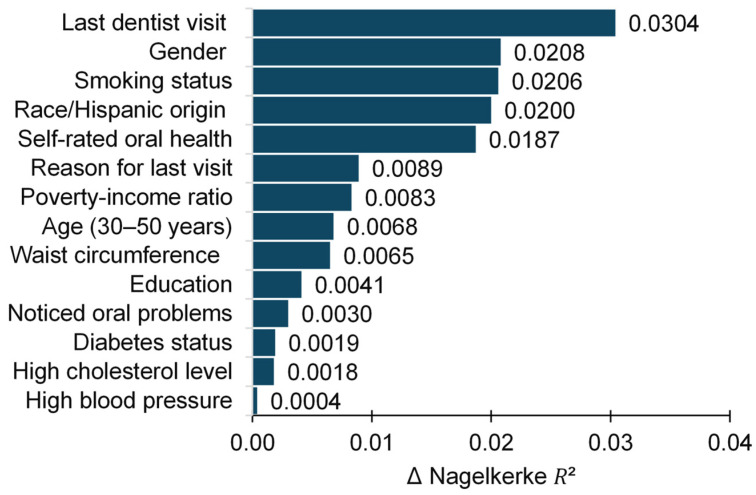
Relative predictor contributions to model explanatory power. Bars show each predictor’s unique contribution to model variance, expressed as the incremental change in Nagelkerke Pseudo-R^2^ (ΔR^2^) within the 14-variable framework. Values represent pooled estimates across 20 imputed datasets using Rubin’s rules. All contributions reflect the predictor effects from the training model used in this study. The total pooled Nagelkerke R^2^ was 0.498. Comparison with the external validation cohort is provided in Supplementary Materials: Table S6.

The model’s predictor importance is established in the training phase (Figure 2), demonstrating a robust Nagelkerke R^2^ of 0.498. When the identical logistic regression framework was refitted using the external validation dataset, it yielded a highly consistent R^2^ value of 0.471 (Supplementary Materials: Table S5). Although minor reconfigurations in predictor importance occurred across cohorts—such as shifts in gender and dental-visit reasons—the overall hierarchy and direction of effects remained stable (Supplementary Materials: Table S6), underscoring consistent performance.

### 3.4. Model Performance, Validation, and Visualisation

External validation using the independent NHANES 2013–2014 cohort (n = 2024) showed strong and clinically meaningful model performance. The model demonstrated a C-statistic (AUC) of 0.822 (95% CI 0.803–0.842). Using the predefined 0.50 cut-off—selected to prioritise clinical utility and case detection (see Supplementary Materials: Note S8)—the model achieved a sensitivity of 73.5% (95% CI 70.7–76.4%) and a specificity of 79.0% (95% CI 76.3–81.7%). The external cohort prevalence (52.1%) fell within expected sampling variation when compared with the total sample prevalence (49.3%). The Brier score in the external cohort was 0.165 (95% CI 0.154–0.176), well below the 0.25 null threshold, indicating good overall predictive accuracy.

**Figure 3:**
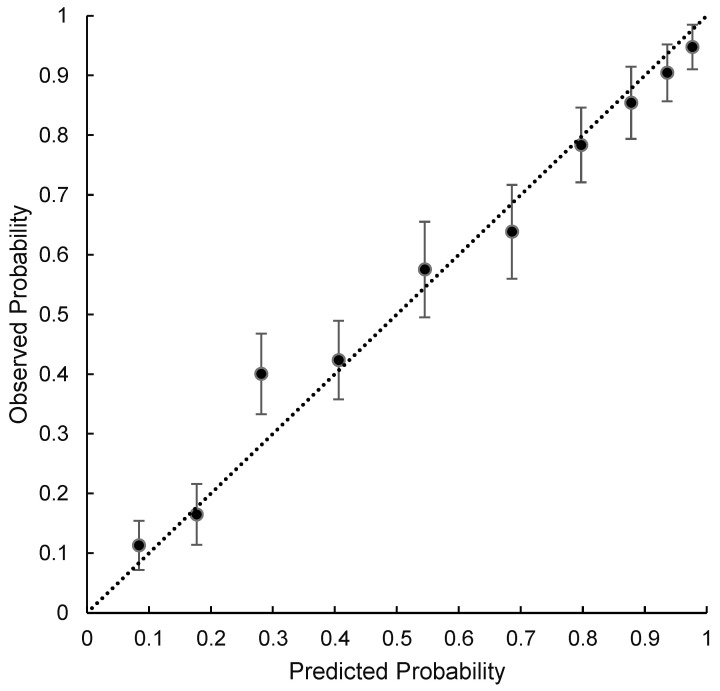
Model calibration (external validation, n = 2024). Points and error bars represent pooled observed vs. predicted risks across deciles (95% CIs via Rubin’s Rules, 20 imputed datasets). The dashed 45-degree line indicates ideal calibration. The pooled slope was 0.85 (95% CI 0.77–0.93) and intercept 0.04 (95% CI −0.08–0.17). While the slope suggests minor overfitting and a localised underestimation occurred at the third decile, the model demonstrated robust calibration across the broader risk spectrum. Internal validation (n = 184) is provided in Supplementary Materials: Figure S2.

External calibration (Figure 3), assessed using multiple imputation (MI) pooling, showed strong agreement across the risk spectrum, with a calibration slope of 0.85 (95% CI 0.77–0.93) and an intercept of 0.04 (95% CI −0.08–0.17). Pooled estimates demonstrated a monotonic increase in predicted risk and near-perfect concordance at higher risk levels, confirming stable performance without evidence of systematic miscalibration.

Internal validation in the hold-out dataset (n = 184) supported these findings. The model achieved an AUC-ROC of 0.817 (95% CI 0.751–0.884). At the same 0.50 threshold, sensitivity was 80.3% (95% CI 71.6–89.0%) and specificity 75.2% (95% CI 65.8–84.6%). The internal cohort Brier score was 0.150 (95% CI 0.118–0.182), consistent with the external results and reflecting the slightly higher prevalence in the hold-out set (53.8%), which aligned with expected stochastic variation.

### 3.5. Sensitivity Analysis and Model Robustness

To assess the influence of multiple imputation (MI), pooled estimates from the primary analysis were compared with a complete-case analysis (CCA; n = 1542) conducted within the training cohort (n = 1686). The model demonstrated high robustness: all 14 predictors retained identical effect directions, and the close agreement in adjusted odds ratio magnitudes supported the plausibility of the Missing At Random assumption (Supplementary Materials: Table S7). Minor shifts in statistical significance—for example, Noticed a tooth that does not look right (*p* = 0.123 in CCA vs. *p* = 0.026 in MI)—were consistent with the loss of efficiency inherent in CCA due to listwise deletion. These differences reflect reduced statistical power rather than changes in the underlying risk structure; whereas CCA discards incomplete observations, MI restores precision by leveraging the full information in the dataset.

Model resilience was further supported by external validation, with a high events-per-variable ratio of 32 (970 events) indicating strong parameter stability. Directional consistency of all predictors was preserved in the external regression model. Only one variable (Main reason for last dental visit) and its category (Planned treatment) showed a more pronounced coefficient shift (0.191 to 0.877). Importantly, this variation did not affect overall discrimination, as global performance metrics remained stable. Full external regression results are provided in Supplementary Materials: Table S5.

### 3.6. Clinical Utility and Net Benefit

**Figure 4:**
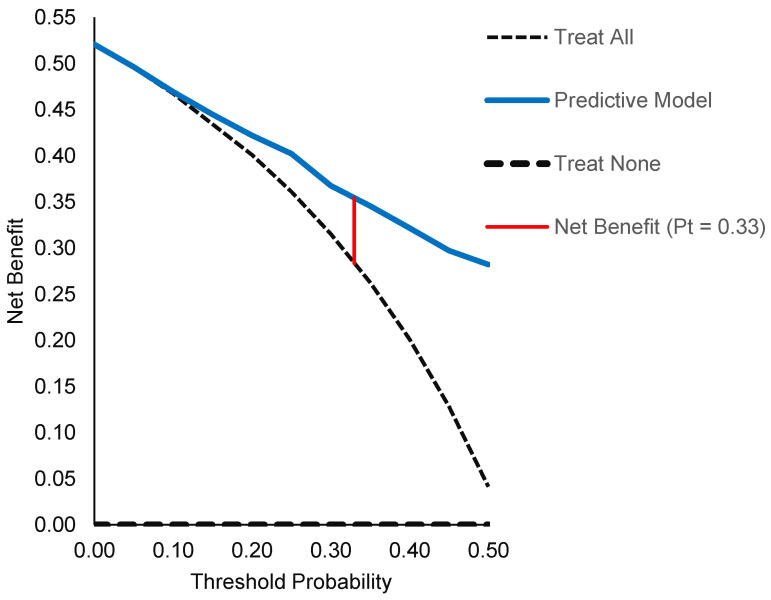
Decision curve analysis in the external validation dataset (NHANES, n = 2024). The model outperformed the treat-all and treat-none strategies across all thresholds. At Pt = 0.33, it yielded a net-benefit gain of 0.070 (~14 fewer unnecessary screenings per 100). Estimates are pooled across 20 imputations; detailed values and internal validation results are provided in Supplementary Materials (Table S9 and Figure S3).

External validation demonstrated clear and clinically meaningful utility of the model. Decision curve analysis (DCA) in the independent NHANES 2013–2014 cohort (n = 2024) showed that the model provided a consistently higher net benefit than both treat-all and treat-none strategies across the full range of threshold probabilities (Pt ≥ 0.00). Detailed threshold-specific net benefit values and confidence intervals are presented in Supplementary Materials (Table S9), with corresponding DCA curves shown in Figure 4.

At the pre-specified clinical threshold of Pt = 0.33—selected to optimise the balance between diagnostic yield and resource allocation—the model achieved a net benefit of 0.354 compared with 0.284 for the treat-all strategy (values for Pt = 0.33 derived via linear interpolation between 0.30 and 0.35). This absolute improvement (ΔNB = 0.070) corresponds to avoiding approximately 14 unnecessary clinical visits per 100 individuals without compromising case detection. The model’s advantage persisted at higher thresholds; for example, at Pt = 0.40, the net benefit was 0.322 versus 0.201 for treat-all.

Further examination of the external cohort showed that the Pt = 0.33 threshold substantially improved sensitivity across multiple clinical domains compared with the conventional Pt = 0.50 cutoff. Sensitivity increased for decayed teeth (90.4% [95% CI 87.5–92.7%] vs. 78.9% [95% CI 75.2–82.3%]) and gum disease (87.1% [95% CI 83.8–89.7%] vs. 79.3% [95% CI 75.6–82.6%]). Similar improvements were observed for oral hygiene and other clinical findings; full subgroup analyses are provided in Supplementary Materials: (Note S9).

Internal validation (n = 184) showed a consistent pattern, with DCA results supporting the findings observed in the external cohort (see Supplementary Materials: Figure S3).

## 4. Discussion

The central finding of this study is the robust and reproducible performance of the prediction model—benchmarked against objective clinical examinations conducted by licensed and calibrated dentists—across consecutive, independently collected NHANES cycles. Discriminative accuracy remained essentially unchanged between the derivation and validation cohorts, demonstrating that the model captures stable, transferable risk signals that consistently emerge across survey waves. The close agreement between two back-to-back nationally representative samples indicates that the model reflects enduring epidemiologic patterns that persist from one cycle to the next.

To assess practical relevance, decision curve analysis confirmed the model’s clinical utility by showing a positive net benefit over a treat-all approach across all thresholds (Pt ≥ 0.00). At the prioritised 0.33 threshold, the model eliminates 14 unnecessary visits per 100 patients while maintaining high sensitivities across various oral health indicators (87.1–95.1%; e.g., 90.4% for decayed teeth) to ensure robust case identification.

The model’s predictive capacity relies on key variables with distinct roles. While the frequency of visits to a dentist served as the primary statistical driver (ΔR^2^ = 0.030), self-assessed teeth and gum health emerged as the superior clinical target. Its utility is demonstrated by a potent maximum effect size (aOR 4.5) and a consistent dose–response gradient, confirmed by external validation. Interestingly, this is further illustrated by large-scale survey data demonstrating that a ‘fair’ or ‘poor’ self-assessment reliably aggregates individuals experiencing persistent physical distress, poor hygiene habits, and socioeconomic disadvantages [24,25]. Congruently, clinical examinations verify that a ‘fair’ or ‘poor’ self-reported rating is consistently associated with a higher prevalence of advanced pathologies like dental caries and periodontitis, validating the biological basis of the patient’s negative self-appraisal [26,27].

Methodologically, the focused 30–50-year age range necessitated conventional weighted regression with normalised weights to maintain model stability, as complex survey models became unstable in sparsely populated strata. The structural reliability of the regression results is evidenced by high EPV ratios (EPV = 27) and stable estimates across multiple imputation and complete-case methods supporting the Missing At Random (MAR) assumption. In addition, notable consistency was found in odds ratios for male gender, borderline diabetes, and intermittent smoking across internal and external cohorts in crude and adjusted results. In contrast, while the effect size for daily smoking decreased in the adjusted model, it remained an important predictor. Additionally, multivariable adjustment revealed important pathways of association and statistical suppression. The attenuation of self-reported diabetes suggests mediation by factors such as waist circumference and race. Furthermore, age and high cholesterol awareness exhibited suppression effects; specifically, adjustment unmasked age as a robust predictor, while high cholesterol awareness showed a protective trend that reached statistical significance in the validation cohort.

The framework’s flexibility was further demonstrated by interchangeability analyses: replacing waist circumference with BMI and substituting self-reported blood pressure with measured metrics proved interchangeable. Moreover, exploratory review indicated that individuals with more pronounced hyperglycemia (e.g., HbA1c ≥ 7.5%) displayed a clear signal, suggesting that metabolic dysregulation—both early and advanced—is a meaningful contributor to the predicted outcome.

By utilising easily accessible health and lifestyle metrics, the model identifies the 30–50-year age range as a strategic window for personalised medicine to intercept declining oral health. In this context, active-matrix metalloproteinase-8 (aMMP-8) levels in mouthrinse offer a promising possibility for enhancing periodontitis screening. As a biomarker, aMMP-8 is well-suited for both personalised medicine and home-based screening, thereby adding new dimensions to predictive modelling [28]. When integrated into dental clinics, chairside aMMP-8 tests equipped with digital readers provide practitioners with high-precision tools for a more objective assessment of disease progression [29]. This integration aligns naturally with clinical practice, as the model developed in this study relies on health and lifestyle data in a way that seamlessly mirrors the standard anamnesis and diagnostic dialogue already performed by dentists.

Furthermore, the model’s external validation yielded a calibration slope of 0.85 (95% CI 0.77–0.93). While a slope below 1.0 typically reflects a slight optimism in the predictor coefficients when applied to a new cohort, this minor variation is highly expected across distinct survey cycles because population characteristics can shift, and individual 2-year NHANES cycles are inherently subject to subset-specific undersampling, as previously noted by Eke et al. [5]. Penalisation techniques (such as Lasso or Ridge) or heuristic shrinkage factors were not applied during model development, as the large sample size and clinical preselection of variables inherently minimised overfitting and the subsequent risk of calibration optimism. Given the strong alignment at the highest risk levels and an intercept close to zero (0.04; 95% CI, −0.08 to 0.17), this minor miscalibration is counterbalanced by the high net benefit demonstrated in the decision curve analysis, suggesting that it is unlikely to impact clinical triage or resource allocation. Nevertheless, a post hoc shrinkage factor can be integrated for operational recalibration to optimise the slope toward 1.0 during digital deployment.

Despite these strengths, several limitations warrant consideration. First, the cross-sectional nature of the NHANES data precludes causal inferences, as the model estimates current need to visit a dentist rather than predicting longitudinal incidence. Second, certain key predictors relied on self-reported data, which may introduce recall or social desirability biases. Moreover, the dataset lacked granular dietary data or validated psychosocial scales. Third, a potential risk of incorporation bias inherent to such designs must be acknowledged. However, as detailed in the Methods section, this risk was structurally minimised through strict procedural, electronic, and conversational blinding within the NHANES protocol [7,12,13]. Ultimately, the robust AUC demonstrated during external validation suggests that these combined data limitations did not critically impair the model’s overall discriminative capacity. Additionally, while the predictors reflect global risk drivers, international variations in healthcare infrastructure and cultural health perceptions necessitate local validation and recalibration prior to international deployment.

To build on these findings, future studies should deploy the model in longitudinal settings to monitor long-term patient flows and project dental care demands. Clinical trials should investigate whether integrating these self-reported predictors into digital primary care health workflows drives patient engagement and timely dental attendance. Comprehensive reviews show that digital tools and mobile health interventions significantly enhance patient self-management, user engagement, and adherence, providing a strong foundation for AI-powered automated triage to optimise these workflows globally across diverse healthcare systems [30,31]. To maintain this high level of performance, proactive refinement—including periodic recalibration—is required to ensure the framework remains optimised for clinical and public health contexts. Similarly, a commitment to robust oversight must be maintained to ensure continuous adherence to all legal and privacy requirements.

## 5. Conclusions

Early midlife (ages 30–50) represents a critical, understudied window for oral health risk stratification. This study addresses this gap through a scalable 14-predictor architecture that delivers stable calibration and substantial clinical net benefit across independent NHANES cycles. Utilising easy-to-use, accessible indicators, the screening framework enables both independent home use by individuals and seamless integration into routine primary or occupational health checks. These consistent risk signals pave a data-driven path toward automated, population-level oral health screening. Building on this validated framework, future geographic and cross-cultural expansion across diverse healthcare systems can optimise resource allocation and enhance targeted, cost-effective interventions to mitigate the global burden of chronic oral diseases.

## Figures and Tables

**Table 1:** Baseline Characteristics of the dentate 30–50-Year-Old NHANES Cohort.

Characteristic	Analytic Sample (n = 1870)	Visit the Dentist (49.3%)	Continue Regular Oral Care (50.7%)
Categorical Variables	Pooled (%)	Pooled (%)	Pooled (%)
Gender			
Female (Ref)	51.4%	42.4%	60.1%
Male	48.6%	57.6%	39.9%
Race/Hispanic origin			
Non-Hispanic white (Ref)	61.7%	50.4%	72.7%
Mexican American	10.6%	15.6%	5.8%
Non-Hispanic black	11.9%	16.8%	7.2%
Other Hispanic	7.2%	9.2%	5.3%
Other Race—Including Multi-Racial	8.5%	8.0%	9.0%
Education			
College graduate or above (Ref)	35.9%	18.3%	52.9%
Some college or AA degree	28.4%	29.6%	27.2%
High school/GED	18.9%	25.5%	12.6%
9–11th (Incl. 12gr. no diploma)	11.6%	18.3%	5.1%
Less than 9th grade	5.2%	8.3%	2.2%
Do you now smoke cigarettes			
Non-smoker (Ref)	77.7%	65.5%	89.5%
Smoker (Some days)	3.9%	5.3%	2.5%
Smoker (Every day)	18.4%	29.2%	8.0%
Doctor told you have diabetes			
No (Ref)	93.7%	91.0%	96.2%
Borderline	1.7%	2.3%	1.1%
Yes	4.6%	6.7%	2.7%
Doctor told you high cholesterol level			
No (Ref)	75.1%	75.2%	74.8%
Yes	24.9%	24.8%	25.2%
Ever told high blood pressure			
No (Ref)	78.3%	72.4%	84.0%
Yes	21.7%	27.6%	16.0%
Rate the health of your teeth and gums			
Excellent (Ref)	11.5%	4.6%	18.2%
Very good	23.3%	13.8%	32.4%
Good	36.0%	33.3%	38.6%
Fair	20.1%	31.5%	9.1%
Poor	9.1%	16.7%	1.7%
When did you last visit a dentist?			
≤6 months (Ref)	43.0%	25.8%	59.8%
>6 to ≤12 months	15.7%	14.4%	17.0%
>1 to ≤2 years	14.4%	18.5%	10.4%
>2 to ≤3 years	7.1%	9.1%	5.1%
>3 to ≤5 years	6.6%	10.6%	2.8%
>5 years or never been	13.2%	21.6%	5.0%
Main reason for last dental visit			
Routine preventive visit (Ref)	64.5%	47.7%	80.8%
Planned treatment	11.0%	14.0%	8.0%
Something was wrong/Never been/other	24.5%	38.2%	11.1%
Noticed a tooth that does not look right			
No (Ref)	84.7%	75.2%	93.9%
Yes	15.3%	24.8%	6.1%
**Continuous Variables**	**Pooled Mean (SD)**	**Pooled Mean (SD)**	**Pooled Mean (SD)**
Age (yrs)	40.2 (6.1)	40.5 (6.0)	39.9 (6.2)
Number of teeth present (1–28)	25.6 (3.8)	24.5 (4.6)	26.6 (2.4)
Ratio of family income to poverty	2.9 (1.7)	2.2 (1.5)	3.6 (1.5)
Waist circumference (cm)	98.6 (15.9)	101.7 (16.4)	95.5 (14.8)

Note: Values represent survey-weighted percentages pooled across 20 multiple imputation cycles (n = 1870). Sampling weights were normalised to the analytic sample size. (Ref) denotes the reference category. Detailed predictor definitions are provided in Supplementary Materials: Table S5. Percentages may not sum to 100% due to rounding.

**Table 2:** Multivariable logistic regression analysis of variables for predicting individual’s need to visit a dentist (Training Set, n = 1686, NHANES cycle 2011–2012).

Predictor Variable	Crude OR (95% CI) ^a^	*p*	B	S.E.	aOR (95% CI) ^b^	*p*
Rate your teeth and gums						
Excellent	1.00	-	-	-	1.00	-
Very Good	1.69 (1.10–2.58)	0.016	0.23	0.25	1.26 (0.77–2.08)	0.356
Good	3.26 (2.20–4.84)	<0.001	0.29	0.24	1.33 (0.83–2.14)	0.232
Fair	12.40 (7.92–19.42)	<0.001	1.12	0.28	3.06 (1.76–5.33)	<0.001
Poor	38.30 (18.40–79.73)	<0.001	1.51	0.43	4.53 (1.94–10.60)	<0.001
Last visit the dentist						
≤6 months	1.00	-	-	-	1.00	-
>6 to ≤12 months	1.91 (1.40–2.61)	<0.001	0.34	0.20	1.40 (0.94–2.08)	0.099
>1 to ≤2 years	4.09 (2.96–5.65)	<0.001	1.17	0.21	3.21 (2.13–4.83)	<0.001
>2 to ≤3 years	4.21 (2.77–6.42)	<0.001	0.85	0.26	2.35 (1.40–3.92)	0.001
>3 to ≤5 years	8.47 (5.24–13.70)	<0.001	1.25	0.29	3.49 (1.97–6.18)	<0.001
>5 years or never	9.37 (6.51–13.49)	<0.001	1.11	0.23	3.02 (1.93–4.71)	<0.001
Main reason for last dental visit						
Routine preventive visit	1.00	-	-	-	1.00	-
Planned treatment	3.25 (2.33–4.55)	<0.001	0.19	0.22	1.21 (0.78–1.88)	0.395
Something was wrong/never been/other	5.75 (4.39–7.55)	<0.001	0.70	0.17	2.02 (1.44–2.84)	<0.001
Noticed a tooth that does not look right						
No	1.00	-	-	-	1.00	-
Yes	5.54 (3.92–7.82)	<0.001	0.52	0.23	1.68 (1.06–2.64)	0.026
Do you now smoke cigarettes?						
Non-smoker	1.00	-	-	-	1.00	-
Smoker (Some days)	3.28 (1.91–5.63)	<0.001	1.21	0.34	3.34 (1.71–6.54)	<0.001
Smoker (Every day)	4.93 (3.66–6.63)	<0.001	1.03	0.20	2.79 (1.89–4.13)	<0.001
Doctor told you have diabetes						
No	1.00	-	-	-	1.00	-
Borderline	2.24 (1.06–4.73)	0.035	0.87	0.47	2.40 (0.95–6.04)	0.064
Yes	2.51 (1.51–4.15)	<0.001	0.09	0.34	1.09 (0.56–2.14)	0.790
Doctor told high cholesterol level						
No	1.00	-	-	-	1.00	-
Yes	0.96 (0.76–1.22)	0.751	−0.28	0.18	0.75 (0.53–1.06)	0.106
Ever told high blood pressure						
No	1.00	-	-	-	1.00	-
Yes	2.05 (1.61–2.62)	<0.001	0.14	0.18	1.15 (0.81–1.63)	0.425
Age (years)	1.017 (1.001–1.033)	0.042	0.041	0.012	1.042 (1.018–1.066)	<0.001
Family income to poverty-ratio	0.59 (0.55–0.63)	<0.001	−0.20	0.05	0.82 (0.74–0.91)	<0.001
Waist circumference (cm)	1.023 (1.016–1.030)	<0.001	0.016	0.005	1.016 (1.006–1.026)	0.001
Gender						
Female	1.00	-	-	-	1.00	-
Male	2.14 (1.75–2.61)	<0.001	0.83	0.14	2.30 (1.76–3.01)	<0.001
Race/Hispanic origin						
Non-Hispanic white	1.00	-	-	-	1.00	
Mexican American	4.34 (3.02–6.24)	<0.001	0.92	0.25	2.50 (1.54–4.04)	<0.001
Non-Hispanic black	3.61 (2.59–5.05)	<0.001	1.04	0.22	2.84 (1.86–4.33)	<0.001
Other Hispanic	2.46 (1.64–3.67)	<0.001	0.68	0.27	1.97 (1.17–3.31)	0.011
Other Race–Incl. Multi-Racial	1.45 (1.01–2.07)	0.042	0.68	0.24	1.98 (1.24–3.16)	0.004
Education						
College graduate or above	1.00 (Ref)	-	-	-	1.00	-
Some college or AA degree	2.99 (2.29–3.90)	<0.001	0.16	0.17	1.17 (0.83–1.65)	0.355
High school/GED	5.61 (4.11–7.66)	<0.001	0.44	0.21	1.56 (1.03–2.35)	0.035
9–11th	9.82 (6.57–14.69)	<0.001	0.62	0.28	1.85 (1.07–3.20)	0.028
Less than 9th grade	11.07 (6.37–19.22)	<0.001	0.49	0.37	1.64 (0.80–3.37)	0.180
Constant	-	-	−5.09	0.70	0.006 (0.002–0.024)	<0.001

Note: n = 1686 (training cohort). Estimates pooled from 20 imputed datasets via Rubin’s Rules. MEC weights were normalised by rescaling them to the analytic sample size. Categorical coding follows Table 1; age, family income-to-poverty ratio, and waist circumference are modeled as continuous variables. B and SE are reported exclusively for the multivariable framework; crude intercepts are omitted to prioritise comparative risk estimates. Abbreviations: B: unstandardised coefficient; SE: standard error; aOR: adjusted odds ratio; CI: confidence interval; *p*: *p*-value. ^a^ Derived from independent bivariate models for each predictor. ^b^ Derived from a single multivariable framework incorporating all 14 predictors simultaneously. Detailed predictor definitions are provided in Supplementary Materials: Table S5.

## Data Availability

The datasets analysed during the current study are available in the NHANES repository.

## Supplementary Materials

The following supporting information can be downloaded at: https://www.mdpi.com/article/10.3390/dj14070398/s1, Note S1: User interaction of the model; Note S2: Robustness of the dependent variable [32,33]; Note S3: Handling missing inputs; Note S4: Internal 90/10 split and rationale [34]; Note S5: Results of model updating; Note S6: Normalisation of weights and sample sizes; Note S7: Prediction model specification and access; Note S8: Cut-off selection for the prediction model; Note S9: Estimating the threshold for DCA [35]; Table S1: Quick estimation guide for the ratio of PIR; Table S2: Missing data patterns (n = 5319); Table S3: Missing data patterns (n = 1907); Table S4: Coding of multivariable model predictors; Table S5: External regression analysis; Table S6: Internal and external Δ Nagelkerke R^2^; Table S7: Imputed model vs. Complete-Case Analysis; Figure S1: Overview of oral health findings; Table S8: Weighted prevalence CIs (Figure S1); Figure S2: Internal calibration plot; Figure S3: Internal DCA curve; Table S9: Net Benefit Values (Internal & External) [36].
