# Supplementary material for: Predicting the Need to Visit a Dentist in Young to Middle-Aged Adults: A Development and External Validation
Source: Dent J (Basel). 2026 Jul 1;14(7):398. doi: 10.3390/dj14070398 (PMC13409249; doi:10.3390/dj14070398)
Supplement: Supplementary file 1 [file dentistry-14-00398-s001.zip › dentistry-4278220-supplementary.pdf]

SUPPLEMENTARY MATERIALS

Manuscript title: Predicting the need to visit a dentist in young to middle-aged adults: a development and external validation

Author and corresponding author: Miika Penttala, Department of Oral and Maxillofacial Diseases, Head and Neck Center, University of Helsinki and Helsinki University Hospital, 00290 Helsinki, Finland; miika.penttala@helsinki.fi

Table of Contents

Section	Content	Page
Note S1	User interaction of the model	S1
Note S2	Robustness of the dependent variable	S2
Note S3	Handling missing inputs	S17
Note S4	Internal 90/10 split and rationale	S20
Note S5	Results of model updating	S23
Note S6	Normalisation of weights and sample sizes	S25
Note S7	Prediction model specification and access	S28
Note S8	Cut-off selection for the prediction model	S29
Note S9	Estimating the threshold for DCA	S31
Table S1	Quick estimation guide for the ratio of PIR	S35
Table S2	Missing data patterns (n = 5,319)	S36
Table S3	Missing data patterns (n = 1,907)	S38
Table S4	Coding of multivariable model predictors	S39
Table S5	External regression analysis	S41
Table S6	Internal and external Δ Nagelkerke R^2	S43
Table S7	Imputed model vs. Complete-Case Analysis	S44
Figure S1	Overview of oral health findings	S46
Table S8	Weighted prevalence CIs (Figure S1)	S47
Figure S2	Internal calibration plot	S48
Figure S3	Internal DCA curve	S49
Table S9	Net Benefit Values (Internal & External)	S49

Supplementary Note S1. User interaction of the model

Users are required to interact with the model primarily during the preparation and verification of input data. Before generating predictions, users must ensure that all required predictor variables are available, correctly formatted, and consistent with the measurement units and coding conventions used during model development. Users may need to review source records, correct miscoded values, or exclude cases with insufficient data quality. No manual tuning of model parameters is required, and the prediction algorithm itself operates automatically once valid input data are provided.

The model is intended for use by individuals with basic competence in handling structured health or survey data. Users should be able to identify missing or implausible values, understand variable definitions, and apply the data-quality checks described in the article, supplementary tables and figures, and notes. Advanced statistical or machine-learning expertise is not required for routine model use, as long as users follow the specified procedures for data preparation and quality assessment.

Users without experience in data management or clinical measurement interpretation should not apply the model without supervision. Incorrect handling of input data may lead to invalid predictions, and therefore adherence to the recommended procedures is essential for safe and appropriate model implementation.

.

Supplementary Note S2. Robustness of the dependent variable: A need to visit a dentist

This note provides a detailed evaluation of the NHANES Overall recommendation for care (OHAREC) variable and its suitability as a dependent binary variable to represent individual's need to visit a dentist. In the NHANES recommendation, examiners assigned each participant to one of four standardised referral categories: (1) see a dentist immediately, (2) see a dentist within 2 weeks, (3) see a dentist at earliest convenience, or (4) continue regular routine care [14]. These four categories were dichotomised in this study, with value of 1 indicating the need to visit a dentist and value of 0 indicating continuation of regular oral care. The variable represents an expert clinical synthesis of the research participant's oral health status.

The Overall recommendation for care (OHAREC) variable is strongly aligned with several underlying NHANES clinical indicators, three of which are Decayed teeth, Gum disease/problem, and Oral hygiene. For the purposes of this note, a fourth variable, Other findings, was created to capture all remaining NHANES oral health findings not included in the other three categories. As expected, having any of these four indicators coded as 1 was strongly associated with a positive value on the Overall Recommendation for Oral Care, which was used in binary form in this study. These four variables—Decayed teeth, Gum disease/problem, Oral hygiene, and Other findings—are described in detail below in their own sections.

For completeness, it is important to note that the procedure described above is highly accurate. Among all participants who had both the predicted outcome and the four variables described above available for analysis, the unimputed dataset of dentate 30–50-year-olds (n = 1,732) showed that only a single participant with a positive value on the predicted outcome (a need to visit a dentist) was not captured by at least one of these four variables and had recorded values that did not indicate a positive finding (this participant was also coded as having no significant

findings in the OHARNF variable; code '1'). In addition, among participants with negative values on the predicted outcome, ten individuals had a value of 1 in at least one of the four indicators described above, which would indicate a need to visit a dentist (all ten had a blank/missing value in the OHARNF variable). Together, these eleven participants accounted for 0.7% of the cohort in the normalised weighted procedure, meaning that 99.3% (95% CI 98.8%–99.6%, n = 1,732) of participants in the predicted outcome (a need to visit a dentist) were correctly identified through the four other NHANES variables described in this note. This level of accuracy was deemed sufficient for the purposes of the present investigation. (The aforementioned 95% confidence interval was calculated using the Wilson score interval with continuity correction.)

For clarity, because of the NHANES coding system, it should be noted that if any of the four variables associated with the predicted outcome had a missing or blank value while the licensed dentist's Overall recommendation of oral care had been recorded, that variable was coded as zero rather than as missing. This approach reflects the fact that the participant had been examined and that the absence of a recorded finding is interpreted as the absence of that condition, provided that the value was not coded as "refused" or "don't know". This rule ensured internal consistency between the clinical indicators and the dentist's overall recommendation.

Each subsection in this note below outlines the construction, epidemiological relevance, and diagnostic performance of the respective variable. In summary, examining these four variables clarifies their role in defining the NHANES Overall recommendation for care variable, which is used in this study in its binary form to predict the need to visit a dentist.

A. Robustness and construction of the Decayed teeth variable (OHAROCDT)

The OHAROCDT variable (Decayed teeth) was evaluated as a proxy indicator of untreated dental caries within the NHANES oral health examination. Its reliability is established by its

consistency with detailed, surface-level clinical examinations performed by trained examiners during the NHANES physical evaluation [32].

Construction of the Decayed teeth variable. The decayed teeth indicator was derived directly from the NHANES oral health examination dataset and recoded for this investigation as follows:

A binary indicator was established where a value of 1 represents the presence of decayed teeth, while a value of 0 was assigned to all other cases, including missing values. To ensure data integrity, decayed teeth values were systematically set to missing in cases where the OHAREC (Overall Recommendation for Care) variable was unavailable. This step was essential to ensure that a "0" value (no decay) was not erroneously assigned to participants who did not have a recommendation completed by a licensed dentist. This rigorous approach was maintained to ensure internal consistency and to eliminate potential misclassification bias resulting from unconfirmed dental health status.

Cohort and Analytical Approach. The performance of the Decayed teeth variable was evaluated in the original, unimputed modeling cohort, consisting of dentate adults aged 30–50 from the NHANES 2011–2012 cycle (n = 1,732). Weighted analyses were performed using normalised NHANES sample weights.

Gold Standard Definition. To identify the presence of dental caries, detailed surface-level clinical data were analysed for all teeth present, excluding wisdom teeth (1, 16, 17, and 32). Each tooth was assessed across multiple surfaces—including occlusal, mesial, distal, buccal, and lingual—to determine its specific condition. This approach allowed for a precise identification of untreated decay by distinguishing active lesions from sound or restored surfaces. A brief methodological note later in this section also comments on the omission of root caries in this cohort and clarifies why this exclusion has only a minimal influence on the predicted outcome.

To ensure diagnostic accuracy, the gold standard for untreated dental caries was defined through a verification process: a case was identified as having untreated dental caries if at least one tooth surface exhibited an active, untreated lesion during a visual-tactile examination. This surface-level clinical finding served as the definitive benchmark against which the binary caries indicator (OHAROCDT) was compared, ensuring that the results reflect pathologically confirmed decay rather than general treatment needs.

Identification accuracy of the decayed teeth variable. The binary Decayed teeth OHAROCDT variable showed excellent agreement with the aforementioned clinical gold standard:

- Matthews Correlation Coefficient: MCC = 0.868 (95% CI 0.856–0.879), n = 1,732.
- Sensitivity: 96.5% (95% CI 94.1%–98.0%), n = 1,732.
- Specificity: 94.4% (95% CI 93.0%–95.5%), n = 1,732.
- Overall Accuracy: 94.9% (95% CI 93.7%–95.9%), n = 1,732.

Note: 95% confidence intervals for sensitivity, specificity, and accuracy were calculated using the Wilson score interval method with continuity correction, while the interval for the Matthews Correlation Coefficient was derived using Fisher's Z-transformation, which stabilises the variance of correlation coefficients.

These results reflect the rigor of the NHANES examination, in which licensed dentists followed NHANES protocols [7,12].

Root caries It is highly unlikely that, within the NHANES 2011–2014 cohort, a participant whose only oral disease or hygiene-related finding is root caries would remain unrecorded in the care-recommendation data. Several clinical, methodological, and structural features of the NHANES protocol make such omission improbable. These considerations are detailed below.

- Mandatory CAL measurement. Clinical attachment loss (CAL) is measured at multiple sites per tooth. This requires placing a periodontal probe directly onto the root surface, making the tactile detection of softened, carious dentin virtually impossible to miss.
- Direct visual exposure. Root caries requires gingival recession. A high CAL value exposes the root surface, placing it directly in the examiner's field of view throughout the assessment.
- General care-recommendation safeguard. Even in survey years without a dedicated root-caries variable, examiners must complete the Recommendations for Care section. Any active root-surface lesion is recorded either as untreated caries or as a general need for dental care.
- Nature of the lesion. Active root caries is clinically conspicuous—typically discolored, softened, and often cavitated. It is not a subtle or subclinical finding and cannot be ethically overlooked.
- Correlation with other findings. Epidemiologically, isolated root caries without concomitant periodontal disease (elevated CAL) or coronal caries is extremely rare. The presence of either condition triggers more detailed examination, increasing the likelihood of detecting root-surface lesions.
- Age-specific considerations (30–50 years). In this age group, root caries is usually secondary to localised periodontitis, trauma, or brushing abrasion. Because these findings are less common than in older adults, they draw examiner attention more strongly.
- Calibration and inter-examiner reliability. NHANES dentists undergo rigorous training and calibration in detecting caries and periodontal disease. This reduces systematic under-reporting and ensures consistent recognition of clinically obvious lesions such as root caries.

- Redundant examination structure. NHANES uses multiple overlapping assessment steps—visual inspection, tactile probing, caries coding, and care-recommendation assignment. A root-surface lesion would need to be missed at every stage, which is highly improbable.
- Standardised examination environment. NHANES assessments occur in controlled conditions without the time pressures of routine clinical practice. Each tooth is examined systematically, increasing detection sensitivity.
- Epidemiological context. Root caries almost always co-occurs with other oral diseases. Root caries as the only finding is a statistically marginal phenotype, making omission even less likely.
- Coding redundancy. Even without a dedicated variable, root caries appears in at least one of three data pathways: untreated caries, care-recommendation coding, or indirectly through CAL/PD findings.
- Ethical obligation. NHANES examiners are licensed dentists with a duty to report all clinically significant findings. Overlooking active root caries would be ethically and professionally unacceptable.

Interpretation. Given its high sensitivity, specificity, overall accuracy, and strong correlation with detailed clinical findings, the Decayed teeth variable functions as a reliable epidemiological screening measure. It effectively condenses complex clinical observations into a binary format suitable for large-scale analyses and for examining associations between oral health and broader health indicators. Importantly, the methodological safeguards described above ensure that the absence of a dedicated root-caries variable does not meaningfully distort the classification of untreated decay or the downstream predictive modeling. The variable therefore provides a stable and interpretable foundation for population-level inference. It was

thereby concluded that the NHANES Decayed teeth (OHAROCDT) variable is an effective and justified factor to predict the outcome of this study: A need to visit a dentist.

B. Robustness and construction of the Gum disease/problem Variable (OHAROCGP)

The Gum disease/problem variable serves as an epidemiological indicator of clinically relevant periodontal involvement. Within the NHANES protocol, licensed dentists perform standardised full-mouth periodontal examinations (FMPE) [7,12]. A positive value is assigned only when the dentist identifies overt clinical signs, such as marked inflammation or clinical attachment loss (CAL) [7,14].

Construction of the Gum disease/problem variable. The Gum disease/problem indicator was derived directly from the NHANES oral health dataset. To ensure consistency with the logic applied in construction of Decayed teeth variable, the variable was coded as follows:

Value = 1 if the NHANES Gum disease/problem variable had a recorded value of 1.

Value = 0 if the variable contained any other value or a missing value.

To safeguard the integrity of the data, a critical exclusion criterion was applied: if the OHAREC (Overall Recommendation for Care) variable was missing, the Gum disease/problem value was systematically set to missing. This step was essential to ensure that a "0" value (no gum disease/problem) was not erroneously assigned to participants who did not have a oral health recommendation completed by a licensed dentist. This rigorous approach was maintained to ensure internal consistency and to eliminate potential misclassification bias resulting from unconfirmed dental health status.

Cohort and analytical approach. The performance of the clinically assessed gum disease/problem indicator was evaluated using the original, unimputed NHANES 2011–2012 dataset. The binary gum disease/problem indicator was validated against clinical gold standards: clinical attachment loss (CAL) and probing depth (PD). These measurements were

obtained from all present teeth, excluding wisdom teeth, in accordance with the NHANES periodontal examination protocol. This clinical validation ensured that the assessed indicator accurately reflects participants' actual periodontal status. For clarity, all of these examination findings—the gum disease/problem indicator as well as the CAL and PD measurements—were recorded during the same NHANES oral health examination.

Only interproximal sites—distal and mesial—were included in the analysis. Mid-buccal and mid-lingual sites were excluded, in line with the diagnostic emphasis on interdental clinical attachment loss described by Tonetti et al. (2018), which prioritises interproximal measurements for periodontitis classification [33].

All participants included in the investigation had complete CAL and PD data. The availability of the clinically assessed gum disease/problem variable (in binary form: 0 = no, 1 = yes) enabled the computation of correlation coefficients, mean-based comparisons, and the examination of CAL-threshold distributions according to the presence or absence of clinically assessed disease/problem. Weighted analyses were performed using normalised NHANES sample weights (n = 1,646).

Association with Clinical Periodontal Measures. The Gum disease/problem variable showed high levels of concordance with established periodontal indicators.

Correlation with clinical attachment loss (CAL):

- A point-biserial correlation analysis revealed a significant positive association between the presence of gum disease and mean CAL (r_{pb} = 0.601 (95% CI 0.569–0.631), n = 1,646). The 95% confidence interval for the point-biserial correlation was calculated using Fisher's z-transformation.

Mean CAL and PD by status:

- The mean CAL was significantly higher in individuals with gum disease or problem (positive individuals value = 1) compared to those without (2.284 mm vs. 1.247 mm,

diff = 1.036 (95% CI 0.954–1.118); $p < 0.001$, Cohen's $d = 1.552$). Similarly, mean Probing Depth (PD) was significantly greater in the disease/problem group (2.109 mm vs. 1.307 mm, diff = 0.802 (95% CI 0.749–0.855); $p < 0.001$, Cohen's $d = 1.770$). Mean differences and p-values were calculated using independent samples t-tests (Welch's t-test due to unequal variances). The observed effect sizes (Cohen's *d*) indicate a very large practical difference between the groups. Statistical significance was defined as $p < 0.001$. n=1,646.

These results demonstrate that individuals identified with gum disease or related problems exhibit considerably higher clinical measurements than those without such findings. The substantially higher clinical attachment loss (CAL) in the affected group—validated through independent clinical examination by licensed dentists—confirms that the variable effectively identifies clinically meaningful periodontal destruction. While CAL can also reflect non-inflammatory recession, the marked intergroup differences suggest that these findings primarily represent pathological attachment loss rather than non-inflammatory mechanical causes.

Diagnostic performance against established definitions. When the Gum disease/problem variable was evaluated against the Tonetti et al. (2018) case definition (CAL ≥ 1 mm) [33]:

- Sensitivity: 100.0% among Gum disease/problem-positive individuals.
- Observation: 100.0% of Gum disease/problem-negative individuals also met the ≥ 1 mm threshold, consistent with the fact that mild attachment loss can occur without active inflammatory disease.

Because the Tonetti et al. (2018) threshold—interproximal CAL ≥ 1 mm at two or more non-adjacent teeth—is easily exceeded in the general population, it fails to meaningfully distinguish healthy from diseased individuals. Consequently, while sensitivity is high, specificity is essentially zero.

When the threshold was increased to CAL ≥ 2 mm,

- 100.0% of the positive group and
- 98.2% of the negative group fulfilled this criterion.

To isolate true inflammatory periodontitis, stricter thresholds were applied:

Methodological details. Distribution of Clinically Significant CAL Thresholds (≥1–5 mm) in the Gum disease/problem variable

CAL threshold	Positive (%)	Negative (%)	Difference (95% CI)	p-value
≥ 1 mm	100.0	100.0	0.0% (–)	–
≥ 2 mm	100.0	98.2	1.8% (0.8–2.9)	< 0.001
≥ 3 mm	96.2	51.9	44.3% (40.7–47.7)	< 0.001
≥ 4 mm	75.9	3.1	72.8% (68.9–76.2)	< 0.001
≥ 5 mm	43.1	0.4	42.7% (38.7–46.7)	< 0.001

Note: This methodological details section compares clinical assessments against specific CAL thresholds. Positive (%): Individuals with a clinically identified gum disease/problem who reached the clinical attachment level (CAL) threshold. Negative (%): Individuals clinically assessed as having no gum disease/problem, yet meeting the CAL threshold. The 95% confidence intervals (CI) for the difference between proportions were calculated using the Newcombe-Wilson method. P-values were calculated using Pearson's chi-square tests. Statistical significance was defined as $p < 0.001$. n = 1,646. CAL, clinical attachment level; CI, confidence interval.

These progressively stricter thresholds demonstrate that the Gum disease/problem -positive group is disproportionately characterised by more advanced stages of attachment loss, whereas such levels are infrequent in the negative group. Because clinically destructive forms of periodontitis in NHANES are represented by the moderate and severe disease categories—which capture the majority of attachment loss and deep periodontal pockets [5]—the distribution indicates that the Gum disease/problem -positive group aligns with more pronounced stages of the periodontal disease spectrum.

Interpretation. Taken together, the substantial correlation between the Gum disease/problem indicator and CAL ($r = 0.60$) is reinforced by the clear clinical separation between groups: Individuals classified as positive exhibited a mean CAL of 2.28 mm and a mean PD of 2.11 mm, whereas those classified as negative showed significantly lower CAL and PD values of 1.25 mm and 1.31 mm, respectively (all $p < 0.001$). Furthermore, the strong separation at higher thresholds—96.2% vs. 51.9% (CAL ≥ 3 mm), 75.9% vs. 3.1% (CAL ≥ 4 mm), and 43.1% vs. 0.4% (CAL ≥ 5 mm)—confirms that the variable effectively isolates clinically significant inflammatory periodontitis ($p < 0.001$ for all comparisons). By distinguishing true periodontal pathology from age-related or mechanically induced attachment loss, the Gum disease/problem variable serves as a robust and practical epidemiological marker.

It was thereby concluded that the NHANES Gum disease/problem (OHAROCGP) variable is an effective and justified factor to predict the outcome of this study: A need to visit a dentist.

C. **Methodological robustness and construction of the Oral hygiene variable (OHAROCOH)**

The OHAROCOH variable (Oral hygiene) was evaluated as an epidemiological indicator of plaque and calculus accumulation within the NHANES oral health examination. NHANES oral health assessments—based on systematic clinical evaluations—have consistently demonstrated high reproducibility and strong inter-examiner reliability within the established calibration framework [7,15]. In this study cohort, the Oral hygiene variable serves as a key clinical indicator reflecting preventive hygiene status and its relationship to broader oral health outcomes.

Construction of the OHAROCOH Variable. The Oral hygiene indicator was derived directly from the NHANES oral health dataset. To maintain consistency with the construction logic used for OHAROCDT and OHAROCGP, the variable was coded as follows:

Value = 1 if the NHANES Oral hygiene variable had a recorded value of 1 (indicating the presence of visible plaque and/or calculus).

Value = 0 if the variable contained any other value or a missing value.

Exception: If the NHANES variable OHAREC (Overall recommendation for care) was missing, the Oral hygiene value was also set to missing, ensuring that the indicator was not assigned when the hygiene examination was incomplete. This rigorous approach was maintained to ensure internal consistency and to eliminate potential misclassification bias resulting from unconfirmed dental health status.

Cohort and Analytical Approach. Building on our previous variable investigations, we assessed the Oral hygiene variable using the same unimputed NHANES 2011–2012 dataset. Weighted analyses were performed using normalised NHANES sample weights. This approach ensures comparability across the different variables studied while accounting for the specific requirements of the current analytical model.

Correlation With Broader Oral Health Indicators. Empirical analysis of the 2011–2012 dataset demonstrated a strong association between oral hygiene and broader oral pathology (Analytic sample size n = 1,732). Within the study cohort, 52.3% (95% CI 48.9%–55.7%) of individuals assessed as needing a visit to a dentist also exhibited poor oral hygiene (Oral Hygiene = 1). Conversely, only 5.2% (95% CI 3.9%–7.0%) of participants with this recommendation presented with poor hygiene as their sole oral health issue (Supplementary Figure S1 and Table S8). Observed proportions are reported as point estimates, with 95% confidence intervals calculated using the Wilson score interval with continuity correction.

This level of association is consistent with the well-documented role of plaque and calculus accumulation as key contributors to both dental caries and periodontal inflammation. The observed relationship confirms that the Oral Hygiene variable functions as a reliable proxy for hygiene-related clinical risk.

Interpretation. The strong correlations observed in this cohort confirm that the Oral hygiene variable is not merely a localised indicator of plaque or calculus but a clinically meaningful proxy for overall oral health status. Its integration into the analytic model ensures that the interplay between preventive hygiene behaviors and clinical disease manifestations is accurately captured.

By reliably identifying individuals with elevated hygiene-related risk, the Oral hygiene variable functions as a robust epidemiological marker and a central component in predicting comprehensive oral health outcomes. It was thereby concluded that the NHANES Oral hygiene (OHAROCOH) variable is an effective and justified factor to predict the outcome of this study: A need to visit a dentist.

D. Methodological context and construction of variable Other findings

The Other findings variable was incorporated into the analytical framework to capture clinically significant pathologies and findings that fall outside the primary categories of dental oral health. In the NHANES oral health examination, these findings represent essential diagnostic markers that trigger professional recommendations for care, ensuring that the composite model reflects the full spectrum of oral morbidity.

Construction of the Other findings Variable. The Other findings variable was derived from supplemental clinical findings documented during the NHANES 2011–2012 oral health assessments. Specifically, the following three variables were combined into a single indicator:

OHAROCCI: Impression of soft tissue condition

OHAROCDE: Denture/Partial Denture/Plates

OHAROTH: Other finding (requiring further follow-up).

To maintain structural consistency with other clinical indicators (Decayed teeth, Gum disease/problem, and Oral hygiene), the variable was coded as a binary measure:

Value = 1: The NHANES examination identified at least one oral health finding in any of the variables OHAROCCI, OHAROCDE, or OHAROTH.

Value = 0: all other cases, including missing values.

Exception: To prevent misclassification, if the NHANES overall recommendation for care (OHAREC) was missing, the composite variable was also set to missing. This rigorous approach was maintained to ensure internal consistency and to eliminate potential misclassification bias resulting from unconfirmed dental health status.

Scope of Clinical Findings. The Other findings variable encompasses various oral health conditions identified during the examination that fall outside the categories of caries or periodontitis. This variable includes:

Soft-tissue abnormalities: Detection of ulcers, suspicious mucosal lesions, or other changes requiring further diagnostic evaluation or biopsy.

Restorative failures: Identification of defective or failing dental restorations that compromise oral function or health.

Trauma and Urgent conditions: Documentation of dental trauma or acute conditions requiring immediate clinical intervention.

Miscellaneous findings: Other documented oral pathologies that triggered a professional recommendation for dental care within the NHANES protocol.

Interpretation. By including the variable Other findings in the dependent variable, the analytical model achieves higher clinical sensitivity. It ensures that individuals whose primary oral health burden arise from mucosal, restorative, or traumatic issues—rather than solely from plaque-related diseases or conditions—are accurately identified.

As a supplemental marker, variable Other findings functions as a necessary component for the holistic representation of oral health status. Its integration ensures that the final composite outcome is not merely a measure of common pathologies, but a robust reflection of all clinically

significant findings documented by the examiners. It was thereby concluded that the Other findings variable is an effective and justified factor to predict the outcome of this study: A need to visit a dentist.

Supplementary Note S3. Handling of missing or poor-quality predictor data during model implementation

This prediction model requires complete and high-quality input data for all 14 predictors included in the final algorithm. To ensure valid and reliable predictions, users should evaluate the availability, plausibility, and consistency of each predictor before applying the model. To ensure the clinical integrity of the prediction, the model is strictly limited to complete-case scenarios. If any of the 14 predictors are missing, the model will not generate an output.

Assessment of data availability and quality

Before generating a prediction, users should verify that:

- All 14 required predictors are present.
- Variables are recorded exactly as defined in Supplementary Table S4 (Operationalisation and coding of multivariable model predictors).
- Categorical variables follow the coding conventions used during model development.
- Continuous variables fall within plausible physiological or measurement-based ranges (detailed outlier thresholds and clinical ranges are provided below).
- Examination values are obtained using methods comparable to those used in NHANES.

Values that are missing, miscoded, or outside plausible ranges should be flagged and reviewed before model use.

Handling of missing predictor values

The model is designed for complete-case application only. Therefore:

- No ad-hoc imputation: Users should not substitute values, carry forward previous measurements, or apply unvalidated imputation procedures.
- Mandatory data: Predictions should only be generated when all 14 predictors are available.

Exclusion: Any case with one or more missing predictors must be excluded from prediction.

Handling of implausible or poor-quality values

To maintain the model's reliability, predictions should not be generated when values fall outside the validated range or are physiologically implausible. The model is strictly validated for:

- Age: 30–50 years.
- Waist circumference: 56.2–176.0 cm, measured to the nearest 0.1 cm at the level of the right iliac crest according to NHANES anthropometry protocols.
- Dentate status: Defined as possessing ≥ 1 erupted permanent tooth (excluding third molars; maximum of 28 teeth).
- Ratio of family income to poverty (range 0–5.0): This continuous value represents the total family income divided by the official poverty threshold for the family size and year (e.g., a ratio of 2.0 indicates income is twice the poverty level). If the exact value is unknown, users can estimate it using the household size and annual income thresholds provided in Supplementary Table S1 (Quick Estimation Guide). In accordance with NHANES confidentiality protocols, all values 5.0 must be capped at 5.0.

Furthermore, predictions should be withheld if:

- A predictor value is physiologically impossible.
- The measurement method (especially for waist circumference) differs substantially from NHANES protocols, which specify measurement to the nearest 0.1 cm at the level of the right iliac crest.
- The value is inconsistent with the model's assumptions (e.g., incompatible units, miscoded categories).

Users should correct such values using source records when possible. If correction is not possible, or the value remains outside the validated boundaries, the case should be excluded from prediction.

Documentation and reporting

Users applying the model in external settings should document:

- The proportion of cases with missing or poor-quality predictor data.
- The criteria used to define implausible or invalid values.
- Any deviations from these recommended procedures.
- The number of predictions withheld due to insufficient data quality or values falling outside the validated ranges (e.g., the applicable age range of 30–50 years).

This documentation supports transparency and helps evaluate the model's applicability in new populations.

Supplementary Note S4. Internal 90/10 split, deterministic assignment, and rationale

To ensure reproducibility and maintain a transparent and prespecified validation structure, the source population was partitioned into a 10% internal validation subset (n = 184) and a 90% model-development cohort (n = 1,686) using a deterministic transformation of the participant sequence number (SEQN). These assignments were held constant across all 20 imputations.

The resulting 90/10 split served only to define a fixed hold-out subset; it was not used for model development, predictor selection, or threshold determination. Instead, 10% internal test cohort functioned solely as a diagnostic check to confirm that the model behaved consistently prior to external validation. Although the split variable was created before imputation, it was not included in the imputation model and therefore did not influence the imputation process. In practical terms, the effective 90/10 split was applied only after imputation. Internal results are reported descriptively in the Results section to demonstrate consistency, but all primary performance inferences and final validation were based on the independent external cohort (n = 2,024).

To support this validation framework, multiple imputation was performed on the full internal dataset to maximise the stability and quality of the imputed values. This approach is consistent with methodological recommendations from TRIPOD, van Buuren, and White et al. [17,18,34], who emphasise that imputation should be performed on the full available dataset to preserve statistical efficiency and avoid unnecessary loss of information. Imputing on the full dataset provides more precise estimates of the underlying distributions and regression relationships than imputing on a reduced training subset. Since the external cohort was fully independent and not involved in the imputation or model development process, this strategy does not introduce bias into the final performance estimates. The external validation closely reproduced the internal C-statistic (AUC) (see Results section in the article), confirming that

the model generalises well and that the imputation strategy did not introduce data leakage or inflate performance.

Fourteen predictors were selected a priori based on their established clinical and epidemiological relevance in previous literature, ensuring that the model development was grounded strictly in theory rather than observed data patterns. No data-driven variable selection procedures were used, which, combined with an events-per-variable (EPV) ratio of 27 (training data), strictly decoupled the model's architecture from both the internal validation subset and the external cohort. This high EPV ratio exceeds commonly recommended thresholds for stable logistic regression, supporting the robustness of the coefficient estimates and ensuring that the model was applied as a fixed, pre-specified algorithm [22]. Consequently, this approach eliminates the risk of overfitting or any inadvertent information transfer between the training process and the independent validation cohorts, as the model performance was evaluated without further optimisation or adjustments.

The final decision threshold (0.50) was chosen based on sensitivity considerations and clinical interpretability, rather than the internal Youden index (0.59) (see Note S8). Thus, neither the internal split nor its performance metrics influenced the operational cut-off or the final model specification. Overall, the methodological choices—including full data imputation, a priori predictor selection, and prioritisation of external validation—were made to ensure that the final model was developed on the highest-quality data available and evaluated in an independent population, consistent with best practices for prediction model research.

To ensure full reproducibility of the imputation process, the SPSS random-number generator was initialised using the command SET SEED = 123456 prior to running the imputations.

The following SPSS syntax was used to implement the deterministic 90/10 assignment based solely on SEQN, ensuring that the same individuals were allocated to the 10% subset across all imputations.

```
* 1. Sort by SEQN to ensure deterministic ordering.
SORT CASES BY SEQN.
* 2. Generate a deterministic pseudo-random value based solely
on SEQN.
COMPUTE RandVal = MOD((SEQN * 9301 + 49297), 233280) / 233280.
* 3. Create the 90/10 split (10% internal validation subset).
COMPUTE Final_Subset = (RandVal < 0.10).
EXECUTE.
```

Supplementary Note S5. Results of model updating and updated model performance

Model updating

This prediction model represents a newly developed model, and no model updating procedures (such as recalibration, coefficient revision, or re-estimation using external data) were performed. All reported results therefore reflect the original model as developed using the NHANES dataset.

Updated model specification

Because no updating was conducted, the final model specification corresponds directly to the development model. The full set of predictor coefficients, intercept, and the coding conventions used for categorical and multi-category predictors are provided in Table 2 in the main manuscript and in Supplementary Tables S2 and S4.

Model performance

All performance metrics reported in the main manuscript represent the performance of the original, unupdated model. These include:

- Discrimination (e.g., C-statistic [AUC])
- Calibration (e.g. calibration plots)
- Overall Performance and Clinical Utility (e.g., sensitivity, specificity, and Decision Curve Analysis [DCA])

No post-update performance metrics are applicable, as the model's original parameters were maintained throughout the evaluation process.

Interpretation

The model's performance has been evaluated through both development and external validation procedures. While the model demonstrated good discriminative ability in both stages, formal calibration assessment (including calibration plotting) was primarily conducted

using the external validation dataset to ensure sufficient sample size and statistical power for reliable estimation. Users should interpret individual risk estimates with caution when applying the model to populations that differ significantly from this validation cohort. Although external validation has been conducted, future geographical or temporal updates may still be necessary to maintain calibration as clinical practices or population characteristics evolve.

Supplementary Note S6. Normalisation of NHANES weights and analytic sample sizes

Survey Weight Normalisation

To ensure valid inference within the restricted analytic cohort (dentate adults aged 30–50 years), survey weights were normalised following established recommendations for subpopulation analyses in complex survey data. Original NHANES MEC weights were included as auxiliary variables during multiple imputation to preserve national representativeness and to reflect the multistage sampling design. After imputation, the sampling weights were rescaled separately within each completed dataset to ensure that the weighted totals remained consistent across imputations. Normalisation w_{norm} was performed by dividing each original MEC weight (w_{MEC}) by the mean weight of the corresponding partition (w_{mean}). The normalised weight is therefore:

$$w_{norm} = w_{MEC}/w_{mean}$$

This rescaling ensures that the weights sum to the analytical sample size, maintaining a stable effective sample size and preventing the artificial inflation of statistical significance.

The analysis initially attempted to follow standard design-based variance estimation using Taylor series linearisation, as described by Wolter [19]. However, due to the restricted subpopulation (dentate 30–50-year-olds) and the low prevalence of certain predictor categories, the original design structure (strata and PSUs) led to computational non-convergence and unstable variance estimation. Consequently, a model-based approach with normalised weights was adopted, consistent with the pragmatic frameworks for complex health surveys proposed by Korn and Graubard [20] and Lumley [21]. This approach ensures that the model estimates remain population-representative and correct for the oversampling design of NHANES while ensuring numerical stability within the multivariable framework.

This procedure anchors the effective sample size to the observed number of cases in the analytic cohort, prevents artificial inflation of statistical significance due to large design weights, and preserves the relative population proportions represented by the original NHANES sampling scheme. All weighting procedures throughout the analysis relied on these normalised weights to ensure methodological consistency across imputed datasets, analytic partitions, and complete-case estimates. Normalisation of survey weights is consistent with recommended practice when analysing subpopulations or domain-specific samples in complex survey designs, particularly when the analytic cohort represents only a subset of the full NHANES sample [19–21].

Participant selection details. Flow of participant selection and analytic sample sizes across all analyses.

**Participant selection**	**Unweighted N**	**Unweighted n**	**Other information**
NHANNES total sample data	9,756		Cycle 2011-2012, Figure 1
Participant exclusion		418	No physical exam
Participant exclusion		4,019	Age <20
Participant exclusion		3,412	Age <30 or >50 years
Participant exclusion		34	Observed edentulism
Participant exclusion		3	Imputed edentulism
**Analyses of imputed data (20 sets)**	**Unweighted pooled n**	**Weighted pooled n (normalised)**	**Other information**
Baseline Characteristics (Internal data)	1,870.20	1,870.20	Table 1
Training sample (Internal data)	1,686.35	1,686.35	Table 2
Test sample (Internal data)	183.85	183.85	Results of the study
Test sample (External data)	2,024.35	2,024.35	Results of the study
Regression (External data)	2,024.35	2,024.35	Supplementary Table S5
**Analyses of non-imputed data**	**Unweighted n**	**Weighted n (normalised)**	**Other information**
A need to visit a dentist variable (Internal data)	1,732	1,732.0	Dependent variable content assessment: Note S2
Decayed teeth variable (Internal data)	1,732	1,732.0	Correlation analysis and calculation of sensitivity: Note S2
Gum disease/problem variable (Internal data)	1,646	1,646.0	Correlation vs CAL, mean-based and sensitivity calculations; Note S2
Oral hygiene variable (Internal data)	1,732	1,732.0	Association estimation: Note S2
Variables comprising dependent variable (Internal data)	1,732	1,732.0	Supplementary Figure S1

Variables comprising dependent variable (External data)	1,855	1,855.0	Supplementary Figure S1
Complete case data (Internal data)	1,542	1,542.0	Regression; Supplementary Table S7
Sensitivity of variables comprising the dependent variable (External data)	1,655	1,655.0	DCA threshold comparison using Complete Case Analysis data; Note S8

Notes:

- Unweighted N = 9,756: The actual number of participants contributing to the analysis.
- Unweighted n: The actual number of unique participants included in each analysis.
- Weighted n (normalised): The sum of NHANES survey weights, normalised to match the specific analytic sample size.
- Analytic sample variation: Sample sizes vary across unimputed analyses due to missing values in specific variables.
- For multiple imputation (20 sets), the reported n represents the average pooled sample size across all sets. These non-integer values arise because the number of individuals excluded due to predicted edentulism varied slightly across the imputation cycles (range: 0–6 exclusions), resulting in minor fluctuations in the analytic sample size (n = 1,867–1,873). Further details on sample size variability and imputation performance are provided in the Results section of the article.

Supplementary Note S7. Full prediction model specification and access

The complete prediction model is fully specified across the main manuscript and the supplementary materials. Predictor definitions, coding rules, regression coefficients, and the model intercept are presented in Table 2 of the main manuscript and in Supplementary Tables S2, S4, and S5. Together, these sources provide all information required for independent implementation of the model in any statistical software environment, including SPSS, R, Python, Stata, and SAS.

Because the model was developed using IBM SPSS Statistics and no proprietary model object or scoring file was generated, no separate model object or syntax file is required for clinical implementation. Predictions can be computed directly by applying the regression equation using the coefficients and intercept reported in the manuscript. The syntax provided in Note S4 is intended solely for the reproduction of the data preprocessing and imputation steps.

Access and reuse: The model specification is freely available for academic and non-commercial use. Users may implement the model without restriction, provided that the original publication is cited. No proprietary components or licensed algorithms beyond standard SPSS procedures were used in model development.

This note therefore confirms that the full model specification is openly available and reproducible based on the information provided in the main article.

Supplementary Note S8: Cut-off selection and performance

Youden-optimal threshold and stability

As an exploratory assessment of threshold behavior, the optimal threshold was calculated for each of the 20 imputed datasets by maximising Youden's Index. The resulting thresholds showed high stability across imputations (mean 0.592, SD 0.013, range 0.572–0.610). Pooled sensitivity and specificity, along with their 95% confidence intervals, were calculated using Rubin's Rules to account for the uncertainty introduced by the multiple imputation process. These analyses were conducted using the internal 10% subset (n = 184) solely to describe threshold characteristics and did not influence model development or the final validation strategy (17).

Rationale for the 0.50 threshold

While 0.592 was the mathematical optimum (e.g., Youden's Index), a threshold of 0.50 was selected for the final model to prioritise clinical utility. Using the internal test dataset (n = 184), this cut-off yielded a sensitivity of 80.3% (95% CI 71.6%–89.0%) and a specificity of 75.2% (95% CI 65.8%–84.6%). These values are presented for descriptive comparison only; the final assessment of threshold performance was based on the independent external cohort. Importantly, the choice of 0.50 was not derived from internal test-set performance but from clinical considerations and was subsequently confirmed—rather than selected—using the external validation cohort (17).

Summary of comparative performance

Sensitivity analyses across an extended probability range confirmed the stability of model discrimination. The 0.50 threshold was identified as the most appropriate cut-off, as it provided an optimal balance between net clinical benefit and the clinical mandate for high sensitivity. For instance, increasing the cut-off to 0.55 resulted in a sensitivity of 78.8% (95% CI 69.8%–87.9%) and a specificity of 76.9% (95% CI 67.8%–86.1%). These comparisons, derived from

the internal subset (n = 184), illustrate threshold trade-offs only; all substantive performance conclusions were drawn from the external validation cohort, which served as the primary and decisive evaluation of model performance (17).

Supplementary Note S9. Estimating the threshold for Decision Curve Analysis (DCA)

Overall threshold selection (Pt = 0.33)

This note details the clinical and analytical rationale for the threshold probability (Pt=0.33) used in this study in the Decision Curve Analysis (DCA). Following the framework established by Vickers, Pt represents the formal trade-off between the harm of unnecessary referrals (false positives) and the risk of undiagnosed disease (false negatives) [35]. In binary prediction models, a threshold of 0.50 is often regarded as the technical standard; however, in a screening context, this default is often too conservative, as it prioritises specificity at the expense of potentially missing a significant number of diseased cases.

When the detection of a disease is prioritised over the avoidance of a single unnecessary clinical encounter, the threshold is appropriately adjusted below the 0.50 standard. In this study, a threshold probability of 0.33 was selected, shifting the focus from a purely technical default to a clinically driven decision-making strategy. This threshold reflects a harm-to-benefit ratio in which identifying one true positive case is valued more highly than avoiding two false positives (1:2 ratio).

To test the model's performance on external data, a sensitivity analysis was conducted among all participants who had both the predicted outcome and all 14 predictors available—that is, a complete-case subset of the unimputed NHANES 2013–2014 dataset of dentate adults aged 30–50 years (n = 1,655). The original model coefficients and intercept were applied to this independent population to evaluate how effectively the 0.33 and 0.50 thresholds identified clinical conditions—such as decayed teeth and periodontal disease/problem—that are included, among others, in the primary outcome (a need to visit a dentist). Findings show that the 0.33 threshold identifies a larger number of individuals classified as having the condition compared with the 0.50 threshold. This reflects the expected increase in sensitivity when applying a lower

decision threshold. The model continues to perform consistently at the conventional 0.50 level, but the 0.33 threshold results in a greater proportion of individuals being flagged as positive.

At the lower threshold (0.33), the model consistently demonstrated higher sensitivity across all diagnostic categories compared with the 0.50 threshold. The improvement was most notable in the detection of decayed teeth, but similar gains were observed for gum disease and related problems, oral hygiene, and other clinical findings. All estimates were based on normalised weighted data, and confidence intervals were calculated using the Wilson score method with continuity correction. Detailed numerical results for each category are presented in the following subsections.

Within this diagnostic framework, the 0.33 threshold functions as an efficient clinical filter. On average, only two additional investigations are required to confirm one true positive case. Furthermore, these clinical encounters carry inherent value beyond diagnosis; even in the absence of disease, they provide definitive rule-out confirmation and patient reassurance. Such proactive engagement is an integral part of a comprehensive care pathway and promotes long-term oral health awareness.

Detailed evaluation of the four components comprising the predicted outcome

The effectiveness of the 0.33 threshold is further demonstrated by its performance across the four aforementioned individual condition components. This uniform threshold ensures a high level of sensitivity while remaining clinically actionable for each specific manifestation.

Decayed teeth. A positive caries-risk classification primarily initiates low-cost, minimally invasive preventive actions, such as counselling, fluoride supplementation, and dietary guidance. Because the clinical cost of a false positive is minimal, a sensitive screening threshold is highly desirable. At the 0.33 threshold, the model achieved a high sensitivity of 90.4% (95% CI 87.5%–92.7%), compared with 78.9% (95% CI 75.2%–82.3%) at the 0.50

threshold, ensuring that the vast majority of individuals in need of preventive caries management are identified early.

Gum disease/problem. The identification of gum disease or related problems initiates a clinical management pathway, which may include periodontal therapy, longitudinal follow-ups, and targeted behavioural interventions. Because these measures require a meaningful allocation of clinical resources, the screening threshold must balance sensitivity with the need to avoid excessive over-referral. At the 0.33 threshold, the model maintained a strong sensitivity of 87.1% (95% CI 83.8%–89.7%), compared with 79.3% (95% CI 75.6%–82.6%) at the 0.50 level, ensuring that clinically significant periodontal destruction is identified early while preserving practical feasibility within routine care.

Oral hygiene. A classification of poor oral hygiene initiates a sustained preventive care pathway, including professional cleanings and behavioural interventions. These strategies require a commitment of clinical resources, making diagnostic stability essential. The model demonstrated strong performance for this component at the 0.33 threshold, with a sensitivity of 89.8% (95% CI 87.0%–92.1%), compared with 79.4% (95% CI 75.9%–82.6%) at the 0.50 level. This indicates that the 0.33 criteria effectively identify individuals requiring enhanced preventive care without a significant loss in performance relative to more conservative thresholds.

Other findings. This category encompasses complex clinical observations, such as soft-tissue abnormalities and prosthesis-related issues, which often trigger specialist referrals or demanding management pathways. The 0.33 threshold proved exceptionally effective for these diverse findings, achieving a sensitivity of 95.1% (95% CI 87.0%–98.5%). Even when evaluated at the more stringent 0.50 level, sensitivity remained high at 88.6% (95% CI 78.9%–94.4%), confirming that the model is reliable for identifying these less common but clinically

significant conditions. See Supplementary Note S2 for further details about the Other findings variable and its construction from NHANES data.

Table S1. Quick Estimation Guide for the Ratio of Family Income to Poverty (PIR). This guide allows users to estimate the PIR value based on total annual household income (gross) and the number of persons living in the household. These thresholds are based on the 2026 U.S. Federal Poverty Guidelines.

Household Size	PIR 1.0 (Poverty Line)	PIR 2.0 (200% of Poverty)	PIR 4.0 (400% of Poverty)	PIR 5.0 (Capped Max)
**1 person**	~$15,960	~$31,920	~$63,840	~$79,800
**2 persons**	~$21,640	~$43,280	~$86,560	~$108,200
**3 persons**	~$27,320	~$54,640	~$109,280	~$136,600
**4 persons**	~$33,000	~$66,000	~$132,000	~$165,000
**5 persons**	~$38,680	~$77,360	~$154,720	~$193,400
**6 persons**	~$44,360	~$88,720	~$177,440	~$221,800
**7 persons**	~$50,040	~$100,080	~$200,160	~$250,200
**8 persons**	~$55,720	~$111,440	~$222,880	~$278,600

Instructions for the User:

1. Select Household Size: Choose the row that corresponds to the number of people living in the household on a shared income.
2. Locate Annual Income: Find the column that most closely matches the total annual gross income (before taxes).
3. Determine the Value:
   - Calculate PIR as income ÷ poverty guideline. Report the value to two decimal places. Example: For a 4-person household, the poverty guideline is $33,000. If the household's annual income is $93,600, then PIR = 93,600 ÷ 33,000 = 2.84.
   - Capping rule: If the total household income is equal to or greater than the PIR 5.0 column, the value 5.0 must be entered into the model.

Table S2. Characteristics and missing data patterns for the imputation sample (NHANES 2011–2012; adults aged 20–80+, dentate and edentulous). Total imputation unweighted sample size: n = 5,319.

Variable name	Role in analysis	Total n	Re-fused (7/77)	Don`t Know (9/99)	Blank/ Missing (.)	Valid n (%)
Gender	IV	5,319	0	0	0	100.0 %
Race/Hispanic origin	IV	5,319	0	0	0	100.0 %
Education	IV	5,319	1	3	0	99.9 %
Self-rated oral health	IV	5,319	0	10	0	99.8 %
When did you last visit a dentist	IV	5,319	0	8	0	99.8 %
Main reason for last dental visit	IV	5,319	0	18	107	97.6 %
Noticed odd looking teeth	IV	5,319	0	5	955	82.0 %
Do you now smoke cigarettes	IV	5,319	3	3	1	99.9 %
High blood pressure	IV	5,319	0	8	0	99.8 %
Doctor told you have diabetes	IV	5,319	0	4	0	99.9 %
High cholesterol level	IV	5,319	0	36	1	99.3 %
A need to visit a dentist	DV	5,319	0	0	462	91.3 %
Poverty-income ratio (PIR)	IV	5,319	0	0	451	91.5 %
Waist circumference (cm)	IV	5,319	0	0	341	93.6 %
Age (years)	IV	5,319	0	0	0	100.0 %
Glycohemoglobin (%)	A	5,319	0	0	299	94.4 %
Permanent tooth present	A	5,319	0	0	389	92.7 %
Total Cholesterol (mg/dL)	A	5,319	0	0	406	92.4 %
Systolic (mm Hg)	A	5,319	0	0	246	95.4 %
Body Mass Index (kg/m2)	A	5,319	0	0	82	98.5 %

Note: Values for 'Refused' and 'Don't know' reflect raw NHANES response codes (e.g., 7 or 9 series). These were treated as missing in the analytic models. Auxiliary variables (e.g., Body Mass Index) were included in the multiple imputation model to satisfy the Missing at Random (MAR) assumption and improve the precision of imputed values.

- Abbreviations: DV: Dependent variable; IV: Independent variable; A: Auxiliary (for MI); PIR: Poverty Income Ratio (Ratio of family income to poverty threshold).
- In the variable When did you last visit a dentist, the NHANES categories More than 5 years ago (6) and Never have been (7) were combined into a single category. In the variable Main reason for last dental visit, there were 107 missing values corresponding

to individuals who had never visited a dentist. These missing values were recoded into a new category, “never been to a dentist”. After this, codes 3, 5, and the new “never been to a dentist” category were merged into one category, and codes 1 and 2 were combined into another category.

- Variable Do you now smoke cigarettes: information from variables SMO020 and SMO040 was combined. Participants who reported smoking fewer than 100 cigarettes in their lifetime were coded as non-smokers, as well as individuals responding not at all. Non-smoker = 1; Smoker (Some days) 2; Smoker (Every day) = 3.
- All combinations of categories for the aforementioned variables were finalised before conducting multiple imputation.
- Number of teeth present was calculated as a sum of individual tooth status variables (OHX02TC–OHX15TC and OHX18TC–OHX31TC). Teeth coding: '1' (present) if the original NHANES code was '2' (permanent tooth present) and '0' otherwise. Third molars (teeth 1, 16,17, and 32) were excluded. Total number of natural teeth was calculated prior to multiple imputation.

Table S3. Characteristics and missing data patterns of study variables and auxiliary variables (NHANES 2011–2012; adults aged 30–50 years, dentate and edentulous). Unweighted sample size: n = 1,907.

Variable Name	Role in analysis	Total n	Re-fused (7/77)	Don`t Know (9/99)	Blank/ Missing (.)	Valid n (%)
A need to visit a dentist	DV	1,907	0	0	141	92.6 %
Age (years)	IV	1,907	0	0	0	100.0 %
Gender	IV	1,907	0	0	0	100.0 %
Race/Hispanic origin	IV	1,907	0	0	0	100.0 %
Education	IV	1,907	0	0	0	100.0 %
Rate the health of your teeth and gums	IV	1,907	0	1	0	99.9 %
When did you last visit a dentist	IV	1,907	0	3	0	99.8 %
Main reason for last dental visit	IV	1,907	0	4	0	99.8 %
Noticed a tooth that doesn't look right	IV	1,907	0	0	1	99.9 %
Do you now smoke cigarettes	IV	1,907	0	2	1	99.8 %
Ratio of family income to poverty (PIR)	IV	1,907	0	0	135	92.9 %
Waist circumference (cm)	IV	1,907	0	0	95	95.0 %
Ever told you had high blood pressure	IV	1,907	0	0	0	100.0 %
Doctor told you have diabetes	IV	1,907	0	3	0	99.8 %
Doctor told you - high cholesterol level	IV	1,907	0	7	1	99.6 %
Glycohemoglobin (%)	A	1,907	0	0	92	95.2 %
Permanent tooth present (No.)	A	1,907	0	0	123	93.6 %
Total Cholesterol (mg/dL)	A	1,907	0	0	121	93.7 %
Systole Blood pressure (mm Hg)	A	1,907	0	0	101	94.7 %
Body Mass Index BMI (kg/m2)	A	1,907	0	0	27	98.6 %

Note: Details of the variables are noted in supplementary Table S2. Abbreviations: DV: Dependent variable; IV: Independent variable; A: Auxiliary (for MI); PIR: Poverty Income Ratio (Ratio of family income to poverty threshold).

Table S4. Operationalisation and coding of dependent and independent variables in multivariable models.

Variable Name	Reference (X=0) vs. Model Indicators (X=1)	Coding Structure
A need to visit a dentist	Continue regular oral care (X=0); A need to visit a dentist (X=1)	0=Continue regular oral care; 1=Visit a dentist
Gender	Female (X=0); Male (X=1)	0=Female; 1=Male
Race/Hispanic origin	Non-Hispanic White (X=0); All other categories (X=1)	1=Non-Hispanic White; 2=Mexican American; 3=Non-Hispanic Black; 4=Other Hispanic; 5=Other Race - Including Multi-Racial
Education	College graduate or above (X=0); All other categories (X=1)	1=College graduate or above; 2=Some college or AA degree; 3=High school graduate/GED or equivalent; 4=9-11th grade (Includes 12th grade with no diploma); 5=Less than 9th grade
Rate the health of your teeth and gums	Excellent (X=0); All other categories (X=1)	1=Excellent; 2=Very good; 3=Good; 4=Fair, 5=Poor
When did you last visit a dentist	6 months or less (X=0); All other categories (X=1)	1=6 months or less; 2=More than 6 months, but not more than 1 year ago; 3=More than 1 year, but not more than 2 years ago; 4=More than 2 years, but not more than 3 years ago; 5=More than 3 years, but not more than 5 years ago; 6=More than 5 years ago or Never have been
Main reason for last dental visit	Category 1 (X=0); All other categories (X=1)	1=Went in on own for check-up, examination, or cleaning and those who were called in by the dentist for check-up, examination, or cleaning; 2=Went for treatment of a condition that dentist discovered at earlier checkup or examination;

		3=Something was wrong, bothering or hurting, or Other, or Never been.
Noticed a tooth that doesn't look right	No (X=0); Yes (X=1)	0=No; 1=Yes
Do you now smoke cigarettes	Not at all (X=0); All other categories (X=1)	1=Not at all (including those who have not smoked at least 100 cigarettes in life);<br>2=Some days;<br>3=Every day
Ever told you had high blood pressure	No (X=0); Yes (X=1)	0=No; 1=Yes
Doctor told you have diabetes	No (X=0); All other categories (X=1)	1=No; 2=Borderline, 3=Yes,
Doctor told you - high cholesterol level	No (X=0); Yes (X=1)	0=No; 1=Yes
Ratio of family income to poverty	-	-
Waist circumference	-	-
Age (years)	-	-

Note: The variable A need to visit a dentist served as the dependent variable (outcome) in all multivariable models presented in this research, including sensitivity analyses. All categorical independent variables were dichotomised; X = 1 indicators were compared against the designated reference category (X = 0) to derive adjusted odds ratios (aORs). Here, "dichotomised" refers specifically to the binary recoding shown in Table S4, not to the original number of categories in the raw NHANES variables. Continuous variables (e.g., age) were modeled linearly and are not displayed in this categorical coding matrix.

Table S5. Multivariable logistic regression analysis of variables for predicting individual´s need to visit a dentist (External dataset, n = 2,024, NHANES cycle 2013–2014)

Predictor Variable	Crude OR (95% CI) [a]	p	B	S.E.	aOR (95% CI) [b]	p
**Rate your teeth and gums**						
Excellent	1.00	-	-	-	1.00	-
Very Good	1.99 (1.32-3.00)	0.001	0.56	0.25	1.74 (1.07-2.84)	0.025
Good	3.15 (2.12-4.69)	< 0.001	0.56	0.24	1.74 (1.08-2.81)	0.023
Fair	12.99 (8.40-20.09)	< 0.001	1.35	0.27	3.86 (2.28-6.53)	< 0.001
Poor	37.15 (19.70-70.04)	< 0.001	1.84	0.37	6.32 (3.04-13.13)	< 0.001
**Last visit the dentist**						
≤6 months	1.00	-	-	-	1.00	-
>6 to ≤12 months	1.62 (1.22-2.16)	< 0.001	0.21	0.19	1.23 (0.85-1.78)	0.272
>1 to ≤2 years	3.25 (2.38-4.43)	< 0.001	0.80	0.20	2.22 (1.51-3.26)	< 0.001
>2 to ≤ 3 years	4.44 (3.06-6.44)	< 0.001	1.02	0.22	2.76 (1.78-4.29)	< 0.001
>3 to ≤ 5 years	5.67 (3.67-8.77)	< 0.001	0.90	0.27	2.46 (1.46-4.17)	< 0.001
>5 years or never	12.32 (8.35-18.18)	< 0.001	1.39	0.24	4.03 (2.54-6,40)	< 0.001
**Main reason for last dental visit**						
Routine Preventive Visit	1.00	-	-	-	1.00	-
Planned treatment	4.01 (2.86-5.63)	< 0.001	0.88	0.21	2.40 (1.58-3.65)	< 0.001
Something was wrong/never been/other	5.58 (4.36-7.14)	< 0.001	0.73	0.16	2.07 (1.51-2.82)	< 0.001
**Noticed a tooth that doesn't look right**						
No	1.00	-	-	-	1.00	-
Yes	6.64 (4.75-9.27)	< 0.001	0.79	0.22	2.20 (1.43-3.38)	< 0.001
**Do you now smoke cigarettes**						
Non-smoker	1.00	-	-	-	1.00	-
Smoker (Some days)	2.84 (1.78-4.53)	< 0.001	0.83	0.30	2.30 (1.27-4.15)	0.006
Smoker (Every day)	4.82 (3.60-6.47)	< 0.001	1.10	0.19	3.02 (2.08-4.37)	< 0.001
**Doctor told you have diabetes**						
No	1.00	-	-	-	1.00	-
Borderline [c]	1.96 (1.03-3.71)	0.039	0.56	0.41	1.76 (0.78-3.95)	0.173
Yes	1.74 (1.17-2.59)	0.007	0.31	0.27	1.37 (0.81-2.32)	0.246
**Doctor told high cholesterol level [d]**						
No	1.00	-	-	-	1.00	-
Yes	0.76 (0.61-0.93)	0.009	-0.37	0.15	0.69 (0.51-0.92)	0.013
**Ever told high blood pressure [e]**						
No	1.00	-	-	-	1.00	-
Yes	1.60 (1.28-1.99)	< 0.001	0.10	0.15	1.10 (0.82-1.48)	0.515
**Age (years)**	1.004 (0.990-1.019)	0.571	0.033	0.010	1.033 (1.012-1.055)	0.002
**Poverty-income -ratio**	0.63 (0.59-0.68)	< 0.001	-0.13	0.05	0.88 (0.80-0.97)	0.007
**Waist circumference (cm)**	1.026 (1.020-1.032)	< 0.001	0.018	0.004	1.018 (1.010-1.026)	< 0.001
**Gender**						

Female	1.00	-	-	-	1.00	-
Male	1.50 (1.25-1.79)	< 0.001	0.36	0.12	1.43 (1.12-1.82)	0.004
**Race/Hispanic origin**						
Non-Hispanic white	1.00	-	-	-	1.00	-
Mexican American	2.79 (2.06-3.78)	< 0.001	0.12	0.21	1.13 (0.75-1.70)	0.557
Non-Hispanic black	2.93 (2.16-3.96)	< 0.001	0.87	0.19	2.40 (1.64-3.50)	< 0.001
Other Hispanic	1.55 (1.06-2.26)	0.023	0.04	0.25	1.04 (0.64-1.68)	0.881
Other Race–Incl. Multi-Racial	1.69 (1.23-2.34)	0.001	1.04	0.21	2.82 (1.85-4.30)	< 0.001
**Education**						
College graduate or above	1.00 (Ref)	-	-	-	1.00	-
Some college or AA degree	2.76 (2.16-3.52)	< 0.001	0.28	0.16	1.32 (0.96-1.82)	0.089
High school/GED	5.82 (4.40-7.71)	< 0.001	0.68	0.19	1.98 (1.37-2.85)	< 0.001
9-11th	8.52 (5.78-12.54)	< 0.001	0.69	0.26	2.00 (1.20-3.33)	0.007
Less than 9th grade	7.49 (4.33-12.95)	< 0.001	0.86	0.37	2.36 (1.15-4.83)	0.019
**Constant**	-	-	-5.00	0.68	0.007 (0.002-0.026)	< 0.001

Note: n = 2,024 (external data); estimates pooled from 20 imputed datasets via Rubin's Rules. MEC weights were normalised by rescaling them to the analytic sample size. Categorical coding follows Table 1 in the article; age, waist circumference, and family income-to-poverty ratio are modeled as continuous variables (see Supplementary Table S1: Quick estimation guide for family income-to-poverty ratio). B and SE are reported exclusively for the multivariable framework; crude intercepts are omitted to prioritise comparative risk estimates. Abbreviations: B: unstandardised coefficient; SE: standard error; aOR: adjusted odds ratio; CI: confidence interval; p: p-value. Definitions: Education categories: AA (associate/vocational), GED (high-school equivalency), 9–11th (incomplete high school), <9th (incomplete middle school). Dental visit: Defined as a visit to a dentist, dental hygienist, or dental clinic (NHANES protocol). Measurements: Waist circumference was measured at the right iliac crest. a Derived from independent bivariate models for each predictor. b Derived from a single multivariable framework incorporating all 14 predictors simultaneously. c Includes prediabetes, impaired fasting glucose, or impaired glucose tolerance as informed by a health professional. d Participant informed by a health professional of having high cholesterol level. e Participant informed by a health professional of having high blood pressure.

Table S6. Predictor-level contributions to model explained variance (Δ Nagelkerke R^2) in the internal (NHANES 2011–2012) and external (NHANES 2013–2014) samples

Rank	Predictor Variable	Internal ΔR^2 ($R^2_{tot} = 0.498$)	External ΔR^2 ($R^2_{tot} = 0.471$)	Change in Rank
1	Last visit to the dentist	-0.0304	-0.0294	0
2	Gender	-0.0208	-0.0038	- 8
3	Do you now smoke cigarettes	-0.0206	-0.0198	0
4	Race/Hispanic origin	-0.0200	-0.0196	0
5	Rate your teeth and gums	-0.0187	-0.0235	+3
6	Main reason for last dental visit	-0.0089	-0.0163	+1
7	Family income to poverty -ratio	-0.0083	-0.0037	-4
8	Age	-0.0068	-0.0047	-1
9	Waist circumference (cm)	-0.0065	-0.0086	+3
10	Education	-0.0041	-0.0082	+3
11	Noticed a tooth that doesn't	-0.0030	-0.0065	+3
12	Doctor told you have diabetes	-0.0019	-0.0014	- 1
13	Doctor told high cholesterol level	-0.0018	-0.0033	+1
14	Ever told high blood pressure	-0.0004	-0.0003	0

Note: Δ Nagelkerke R^2 represents the unique contribution of each variable to the total model variance, calculated by the incremental change in the pseudo-R^2 value. This incremental change was obtained by removing each predictor from the full model and computing the reduction in pseudo-R^2. Change in Rank reflects the shift in the relative importance of predictors—determined by their unique Δ Nagelkerke R^2 contribution—when comparing the internal model (baseline) to the external regression. A positive value (+) indicates that a variable climbed in importance in the external model, a negative value (–) indicates a decrease in relative rank, and zero (0) denotes a stable rank across both models. Pooled estimates are derived from 20 multiple imputations using Rubin's Rules. Internal data: n = 1,686; external data: n = 2,024.

Table S7. Sensitivity Analysis: Comparison of Complete-Case Analysis vs. Primary Imputed Model (NHANES 2011–2012)

Predictor Variable	Complete-Case (CCA) (n = 1,542)	Primary Model (MI) (n = 1,686)	Directional Consistency
**Rate your teeth and gums**			
Excellent	1.00	1.00	-
Very Good	1.04 (0.63-1.72)	1.26 (0.77-2.08)	Consistent
Good	1.25 (0.78-2.02)	1.33 (0.83-2.14)	Consistent
Fair	3.03 (1.73-5.27)***	3.06 (1.76-5.33)***	Consistent
Poor	3.93 (1.74-8.87)***	4.53 (1.94-10.60)***	Consistent
**Last visit to the dentist**			
≤6 months	1.00	1.00	-
>6 to ≤12 months	1.24 (0.84-1.81)	1.40 (0.94-2.08)	Consistent
>1 to ≤2 years	3.67 (2.44-5.51)***	3.21 (2.13-4.83)***	Consistent
>2 to ≤ 3 years	2.22 (1.32-3.72)**	2.35 (1.40-3.92)**	Consistent
>3 to ≤ 5 years	4.25 (2.32-7.77)***	3.49 (1.97-6.18)***	Consistent
>5 years or never	3.24 (1.99-5.27)***	3.02 (1.93-4,71)***	Consistent
**Main reason for last dental visit**			
Routine Preventive Visit	1.00	1.00	-
Planned treatment	1.07 (0.68-1.68)	1.21 (0.78-1.88)	Consistent
Something was wrong/never been/other	2.31 (1.64-3.26)***	2.02 (1.44-2.84)***	Consistent
**Noticed a tooth that doesn't look right**			
No	1.00	1.00	-
Yes	1.43 (0.91-2.25); *p*=0.123	1.68 (1.06-2.64)*	Consistent
**Do you now smoke cigarettes**			
Non-smoker	1.00	1.00	-
Smoker (Some days)	2.51 (1.30-4.84)**	3.34 (1.71-6.54)***	Consistent
Smoker (Every day)	3.12 (2.09-4.66)***	2.79 (1.89-4.13)***	Consistent
**Doctor told you have diabetes**			
No	1.00	1.00	-
Borderline	2.22 (0.84-5.86)	2.40 (0.95-6.04)	Consistent
Yes	1.40 (0.71-2.77)	1.09 (0.56-2.14)	Consistent
**Doctor told high cholesterol level**			
No	1.00	1.00	Consistent
Yes	0.77 (0.55-1.07)	0.75 (0.53-1.06)	Consistent
**Ever told high blood pressure**			
No	1.00	1.00	-
Yes	1.15 (0.81-1.62)	1.15 (0.81-1.63)	Consistent
**Age (years)**	1.047 (1.023-1.071)***	1.042 (1.018-1.066)***	Consistent
**Family income to poverty -ratio**	0.80 (0.72-0.88)***	0.82 (0.74-0.91)***	Consistent

**Waist circumference (cm)**	1.020 (1.010-1.030)***	1.016 (1.006-1.026)**	Consistent
**Gender**			
Female	1.00	1.00	-
Male	2.29 (1.74-3.01)***	2.30 (1.76-3.01)***	Consistent
**Race/Hispanic origin**			
Non-Hispanic white	1.00	1.00	-
Mexican American	2.42 (1.47-3.98)***	2.50 (1.54-4.04)***	Consistent
Non-Hispanic black	2.94 (1.89-4.58)***	2.84 (1.86-4.33)***	Consistent
Other Hispanic	2.19 (1.29-3.73)**	1.97 (1.17-3.31)*	Consistent
Other Race – Incl. Multi-Racial	2.06 (1.27-3.36)**	1.98 (1.24-3.16)**	Consistent
**Education**			
College graduate or above	1.00	1.00	-
Some college or AA degree	1.19 (0.85-1.67)	1.17 (0.83-1.65)	Consistent
High school/GED	1.44 (0.96-2.17)	1.56 (1.03-2.35)*	Consistent
9-11th	2.03 (1.15-3.60)*	1.85 (1.07-3.20)*	Consistent
Less than 9th grade	1.53 (0.69-3.50)	1.64 (0.80-3.37)	Consistent

Note: Normalised survey weights were applied independently within both CCA and MI frameworks to account for the respective data structures. * $p < 0.05$, ** $p < 0.01$, *** $p < 0.001$. CCA = complete-case analysis; MI = Multiple Imputation.

A. Overall prevalence of findings

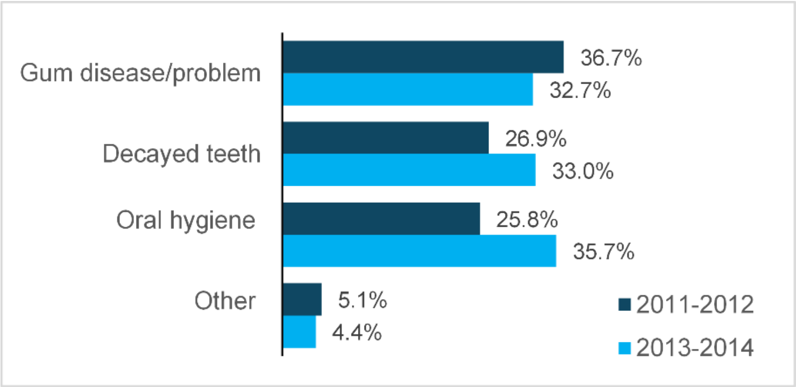

B. Prevalence of isolated findings

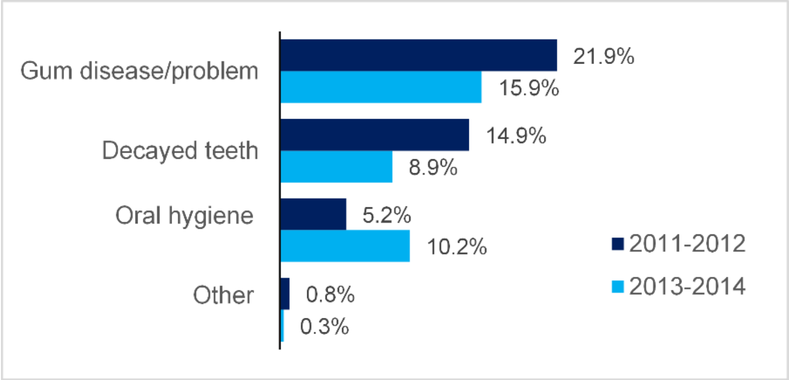

C. Prevalence of the findings in the positive outcome variable

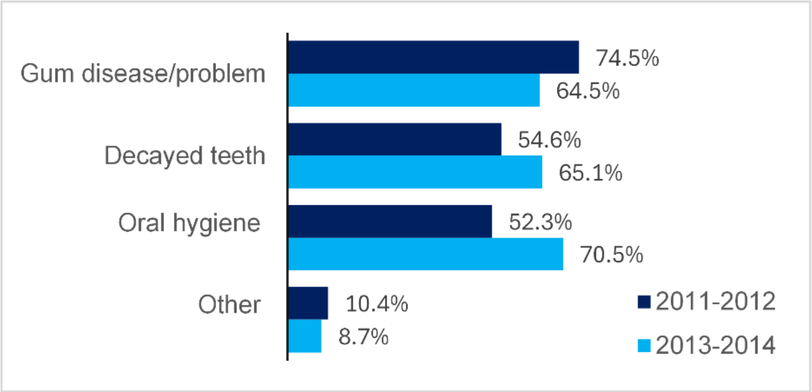

Supplementary Figure S1. Overview of oral health findings in NHANES 2011–2012 and 2013–2014 cycles among dentate adults aged 30–50 years. (A) prevalence in the total population, (B) prevalence of isolated findings, and (C) prevalence of findings within the positive outcome variable (a need to visit a dentist). Data were assessed using unimputed datasets. Analytic sample sizes: internal n = 1,732; external n = 1,855. Weighted analyses were performed using normalised NHANES sample weights. An isolated finding is defined as a

condition occurring independently of the other included clinical parameters shown in the corresponding figures. Corresponding numerical estimates are provided in Table S8 immediately below.

Table S8. Weighted 95% confidence intervals (CI) corresponding to the prevalence rates shown in Supplementary Figure S1 for the internal and external cohorts.

Finding	Cohort	Total Prevalence 95 % CI	Prevalence (isolated) 95 % CI	Outcome Prevalence 95 % CI
Gum disease/problem	Internal	34.4%–39.0%	19.2%–24.8%	71.4%–77.4%
	External	30.6%–34.9%	13.7%–18.5%	61.3%–67.5%
Decayed teeth	Internal	24.8%–29.1%	12.6%–17.5%	51.2%–58.0%
	External	30.9%–35.2%	7.2%–10.9%	62.0%–68.1%
Oral hygiene	Internal	23.7%–27.9%	3.9%–7.0%	48.9%–55.7%
	External	33.6%–38.0%	8.4%–12.4%	67.4%–73.4%
Other	Internal	4.1%–6.3%	0.3%–1.7%	8.4%–12.7%
	External	3.5%–5.5%	0.1%–1.0%	7.0%–10.7%

Note: All 95% CI estimates are based on normalised weighted data. Outcome prevalence refers to findings within the positive outcome variable (a need to visit a dentist). The 95% confidence intervals were calculated using the Wilson score interval with continuity correction. Although statistically significant differences in prevalence rates were observed between cohorts, the outcome they collectively comprise—the need to visit a dentist—did not differ significantly between the cohorts, as assessed using the Newcombe–Wilson method (prevalence difference between the 2011–2012 and 2013–2014 cohorts: −1.43%, 95% CI −4.73%–1.89%; $p = 0.39$). The p-value was calculated using Pearson's chi-square test.

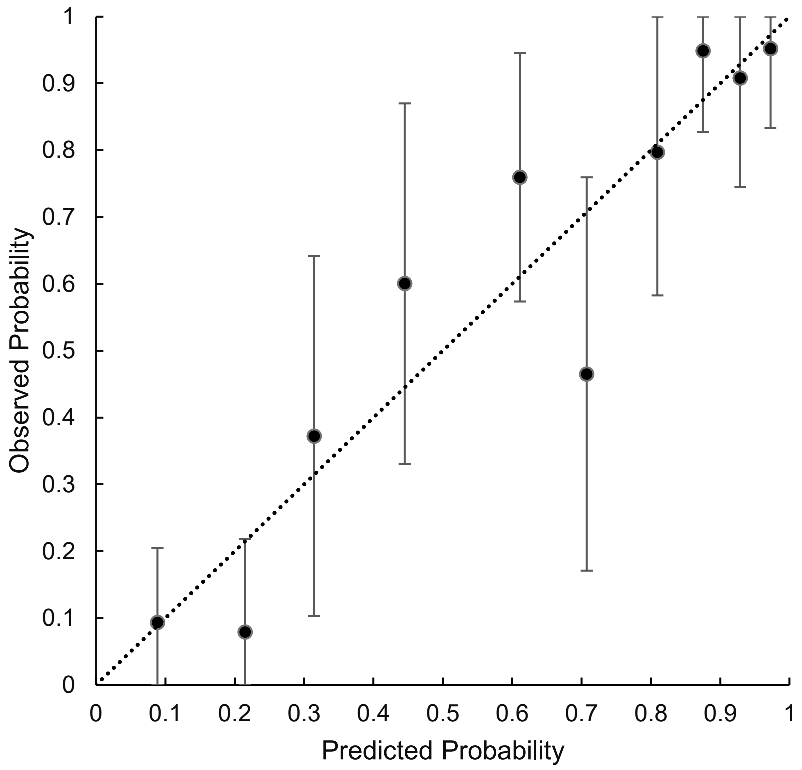

Supplementary Figure S2. Internal calibration plot (n = 184). The pooled calibration slope was 1.03 (95% CI 0.72–1.34) and the intercept was 0.03 (95% CI -0.38–0.45), indicating strong overall calibration in the development cohort. The dashed line represents perfect calibration (identity line), while points and error bars represent pooled observed probabilities against predicted probabilities across deciles. Predicted and observed outcomes show close agreement across the entire risk spectrum; although slight fluctuations are visible, all 95% confidence intervals (CIs) encompass the identity line. Calibration is particularly precise in the upper deciles, where predicted and observed risks are nearly identical. Error bars represent 95% CIs calculated using Rubin's rules across 20 imputed datasets.

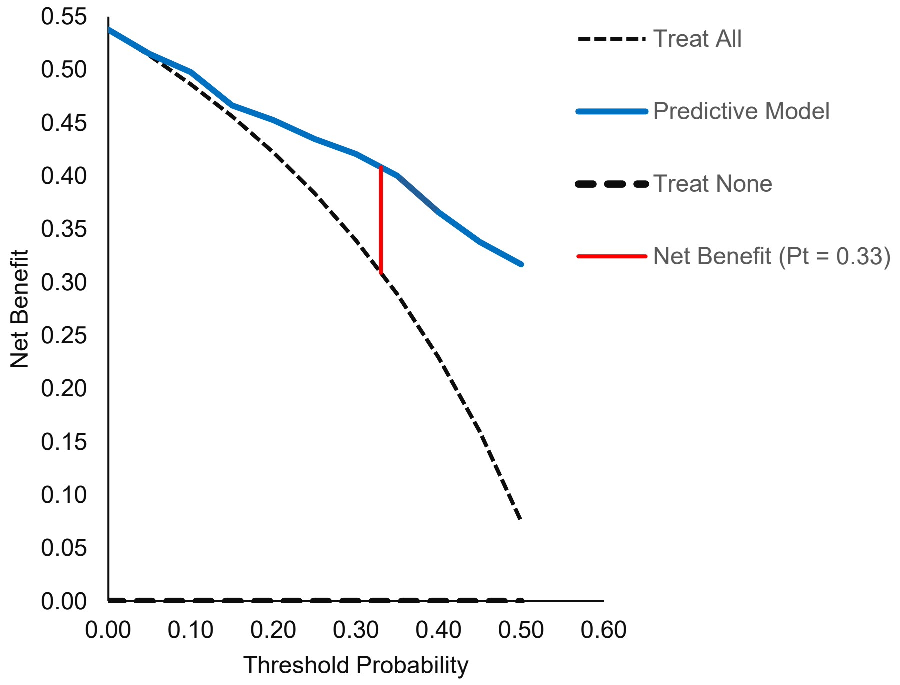

Supplementary Figure S3. Decision curve analysis (DCA) for the prediction model in the internal validation dataset (n = 184). Internal validation showed a consistent pattern where the model surpassed the treat-all strategy across the relevant clinical range. At the clinically selected threshold (Pt = 0.33), the model provided a pooled net benefit of 0.408 compared to 0.309 for the treat-all strategy, representing a 0.099 higher net benefit. This superiority was maintained at higher thresholds; for example, at Pt = 0.40 the model's net benefit was 0.366 versus 0.230 for treat-all. The consistency between internal and external validation curves (Figure 4 in the main article) indicates robust decision-analytic performance, highlighting the model's potential to optimise resource allocation in primary care and population-level screening programs. Pooled estimates and confidence intervals were derived from 20 imputed datasets using Rubin's rules. Corresponding numerical estimates are presented in Table S9 immediately below.

Table S9. Decision Curve Analysis: Net Benefit for Internal and External Validation Datasets. The table presents the clinical net benefit of the 14-predictor model compared to a treat-all strategy across a range of threshold probabilities (Pt). Results are provided for the 2011–2012

internal validation dataset (upper section) and the 2013–2014 external validation dataset (lower section). Net benefit values represent pooled estimates across 20 multiple imputed datasets to account for missing data, with 95% confidence intervals (CI) calculated using Rubin's rules. A model is considered clinically useful at a given threshold if its net benefit is both positive and higher than the treat-all strategy.

**Threshold (Pt)**	**Pooled Treat All (Average of 20 MI)**	**95 % CI (Lower – Upper)**	**Pooled Net Benefit (Average of 20 MI)**	**95 % CI (Lower – Upper)**
**Internal data n = 184**				
0.00	0.538	0.462 – 0.614	0.538	0.462 – 0.614
0.05	0.513	0.433– 0.594	0.515	0.435– 0.595
0.10	0.486	0.402 – 0.571	0.498	0.413 – 0.583
0.15	0.456	0.367 – 0.546	0.466	0.375 – 0.558
0.20	0.422	0.327 – 0.517	0.453	0.356 – 0.549
0.25	0.384	0.282 – 0.485	0.435	0.332 – 0.537
0.30	0.340	0.231– 0.448	0.421	0.309 – 0.532
0.35	0.289	0.172 – 0.406	0.400	0.279 – 0.521
0.40	0.230	0.103 – 0.357	0.366	0.235 – 0.496
0.45	0.160	0.021 – 0.298	0.338	0.195 – 0.481
0.50	0.076	-0.077 – 0.228	0.317	0.160 – 0.474
**External data n = 2,024**				
0.00	0.521	0.497 – 0.544	0.521	0.497 – 0.544
0.05	0.495	0.471 – 0.520	0.496	0.471 – 0.521
0.10	0.467	0.441 – 0.494	0.469	0.443 – 0.496
0.15	0.434	0.408 – 0.464	0.445	0.417 – 0.473
0.20	0.401	0.371 – 0.430	0.422	0.392 – 0.452
0.25	0.361	0.329 – 0.392	0.402	0.370 – 0.434
0.30	0.315	0.281– 0.349	0.367	0.333 – 0.402
0.35	0.263	0.226 – 0.299	0.345	0.308 – 0.383
0.40	0.201	0.162 – 0.240	0.322	0.281 – 0.362
0.45	0.128	0.086 – 0.171	0.297	0.253 – 0.341
0.50	0.041	-0.006 – 0.088	0.282	0.234 – 0.330

Note: Across the entire range of threshold probabilities (Pt) from 0 to 0.50, the model consistently provides a higher point estimate for net benefit compared to the treat-all strategy in both the internal and external validation datasets. In the external validation, a marginal overlap (0.318 vs. 0.319) is observed between the individual 95% CIs at the specific threshold of 0.33, obtained via linear interpolation of Pt values within the 0.30–0.35 range. This represents an overly conservative measure of uncertainty resulting from the use of the Delta

method to calculate standard errors for net benefit. While the Delta method provides a robust and unified statistical framework, comparing individual CIs ignores the inherent correlation between Net Benefit estimates (e.g., model-based vs. Treat All) derived from the same cohort. Consequently, as noted by Vickers et al. (2023) [36], comparing individual CIs in this manner is an invalid method for formal hypothesis testing. Despite this marginal overlap at a single point, the substantial point estimate advantage (0.070) and the consistent overall trend across the threshold range strongly support the statistical superiority and clinical utility of the model (see Results section in the main article).
